# Development & assessment of polyherbal extracts for treating oral cancer by integrating phytochemistry, bioactivities, and network pharmacology

**DOI:** 10.1038/s41598-026-53348-z

**Published:** 2026-07-10

**Authors:** E. Jancy Mary, L. Inbathamizh, Vinay Kumar Pandey, G. Shoba, Shailendra Thapliyal, Ayaz Mukarram Shaikh, Béla Kovács

**Affiliations:** 1 Faculty of Science (Life Sciences), Sathyabama Institute of Science and Technology, Chennai, 600119 Tamilnadu India; 2 Department of Biotechnology, Sathyabama Institute of Science and Technology, Chennai, 600119 Tamilnadu India; 3 Department of Food Science and Technology, Graphic Era (Deemed to be University), Dehradun, Uttarakhand India; 4 Department of Biotechnology, Dwaraka Doss Goverdhan Doss Vaishnav College, Chennai, 600106 Tamilnadu India; 5 Uttaranchal Institute of Technology, Uttaranchal University, Dehradun, 248007 Uttarakhand India; 6 Faculty of Agriculture, Food Science & Environmental Management, Institute of Food Science, University of Debrecen, Böszörményiút 138, Debrecen, 4032 Hungary

**Keywords:** Polyherbal extract, Bioactivities, Oral squamous carcinoma, UPLC, Network pharmacology, Biochemistry, Cancer, Computational biology and bioinformatics, Drug discovery, Plant sciences

## Abstract

Oral carcinoma is a major global health concern and a leading cause of carcinoma-related mortality. This study evaluated a methanolic polyherbal extract for its phytochemical composition and biological efficacy, along with mechanistic insights via in silico network pharmacology. GC-MS and UPLC profiling confirmed diverse bioactive compounds, particularly polyphenols, with UV peaks at 272 nm. The extract showed strong antioxidant activity (IC₅₀ = 108 ± 0.17 µg/mL) and significant antimicrobial effects (inhibition zones: 6.00–21.33 mm). Cytotoxicity assays revealed anticancer activity against OECM-1 oral carcinoma cells (IC₅₀ = 18.86 µg/mL). The polyphenolic fraction suppressed pro-inflammatory cytokines (IL-6, IL-10, TNF-α, IL-1β, TGF-β) with IC₅₀ values of 3.02–6.15 µg/mL. Network pharmacology and molecular docking indicated multi-target interactions of key phytochemicals in pathways relevant to oral carcinoma progression. These findings suggest the polyherbal extract may be a complementary candidate in oral carcinoma management via antioxidant, anti-inflammatory, and antiproliferative effects.

**Supplementary Information:**

The online version contains supplementary material available at 10.1038/s41598-026-53348-z.

## Introduction

Plants have long been fundamental sources of medicinal compounds, rich in bioactive metabolites like polyphenols, flavonoids, alkaloids, and terpenoids, these formulations exhibit potent antioxidant, antimicrobial, and anti-inflammatory properties, making them attractive for cost-effective treatments in resource-limited settings^1,2^. OECM is a oral squamous cell carcinoma (OSCC), a significant subtype of oral cancer, is intimately associated with oxidative stress, microbial infections, and inflammation. The accumulation of reactive oxygen species (ROS), activation of pro-inflammatory mediators such as COX-2, and microbial pathogens like *Streptococcus mutans*,* Staphylococcus aureus*,* Actinomyces spp.*, and *Candida albicans* exacerbate oral disease and cancer risk^3–5^. Phytochemicals such as flavonoids, tannins, and phenolics exhibit antioxidant and antimicrobial effects while modulating inflammatory pathways, thus addressing multiple contributors to oral carcinogenesis^6–8^. Moreover, polyherbal combinations often outperform individual plant extracts due to synergistic activity^3,8^. Modern analytical tools, including Gas Chromatography-Mass Spectrometry (GC-MS), Ultra-Performance Liquid Chromatography (UPLC), and Nuclear Magnetic Resonance (NMR), have enhanced the profiling of plant-derived bioactive compounds, enabling comprehensive phytochemical characterization^9–11^. These methods provide structural insights into bioactive molecules and link them to biological activities. Given the limitations of conventional oral carcinoma therapies marked by poor survival rates and high toxicity. Polyphenols have gained attention for their multitarget actions, including ROS scavenging and inhibition of tumour proliferation^12^. Network pharmacology offers a systems-level approach to understand the multi-component, multi-target mechanisms of herbal formulations, integrating molecular interactions and pathway analysis to guide drug discovery^13,14^.

Oral squamous cell carcinoma remains a severe health burden due to toxicity, resistance, and poor long-term outcomes. Limited phytochemical characterization and mechanistic insight impede the translation of polyherbal bioactive into evidence-based oral cancer treatments. This study links traditional polyherbal knowledge and molecular oncology by merging experimental validation with network pharmacology to build a mechanism-driven, evidence-based framework for oral carcinoma therapy.

## Methodology

### Assemblage and authentication of medicinal herbal plants

Medicinal plants including *Plectranthus amboinicus*, *Ocimum tenuiflorum*, *Mentha piperita*, *Trigonella foenum-graecum*, and *Azadirachta indica* were collected from local sources in Chennai, India. The identity and authenticity of the plant species were confirmed by Dr. P. Sathiyarajeswaran (Assistant Director in-charge) and Dr. K. N. Sunil Kumar (Head, Department of Pharmacognosy), Central Council for Research in Siddha (CCRS), Arumbakkam, Chennai, by evaluating both morphological and microscopical characteristics. A certificate of authentication was issued under reference number: 252−06082101-05.

### Preparation of plant extracts

The collected plant materials were washed thoroughly with tap water, shade dried for 25–30 days, and then coarsely powdered. Equal quantities of the powdered leaves from each plant were combined to form a polyherbal blend. For extraction, 10 g of the polyherbal powder was subjected to successive solvent extraction using methanol, acetone, ethyl acetate, chloroform, and water in a conical flask. The mixture was agitated for 72 h using an orbital shaker. The extraction was repeated thrice using fresh solvent each time. The filtrates were evaporated at ambient temperature to remove the solvent, and the resulting extracts were dissolved in their respective solvents to obtain a stock solution of 10 mg/mL. The dried extracts were stored in airtight containers until further use for phytochemical and biological analyses^15,16^.

### Phytonutrient screening

Preliminary phytochemical screening of the polyherbal extract was conducted to identify bioactive constituents such as sugars, polypeptides, amino acids, saponins, tannins, flavonoids, alkaloids, glycosides, polyphenols, terpenoids, and fatty acids using standard qualitative methods with minor modifications. Qualitative assays were conducted at room temperature (25 ± 2 °C) using extracts produced at a concentration of 10 mg/mL^17–19^.

### Phytochemical compounds by GC/MS evaluation

The methanolic formulation of the polyherbal mixture was subjected to Gas Chromatography–Mass Spectrometry (GC/MS) analysis to identify and confirm its phytochemical constituents. The analysis was performed using an Agilent 8890 GC system with a mass detection range of 50–600 m/z and a scan speed of 1562 (*n* = 2). GC/MS was conducted using a fused silica capillary column (30 m × 250 μm × 0.25 μm). The maximum oven temperature was set to 350 °C with a 1-minute equilibration time.

A high-purity monatomic gas served as the carrier, with a flow rate of 0.0012 L/min. The split ratio was maintained at 5:1 using a 10 µL syringe. The ionization was carried out using electron impact (EI) mode at 70 eV. The total run time for the GC/MS analysis was 3210 s.

The chromatographic data provided information on phytonutrient identity, retention time, peak area percentage, molecular formula, and molecular mass^20–23^. Compound identification was achieved by comparing the acquired mass spectra with those in the NIST (National institute of standards and technology) mass spectral library. Unknown spectra were matched with known reference spectra to determine compound identity and chemical characteristics.

### Phytochemical compounds by UPLC analysis

Phytochemical profiling of the polyherbal formulation was performed using an ACQUITY UPLC system (Waters) coupled with a Xevo G2-XS QTof mass spectrometer. The UPLC setup consisted of a column unit, sample manager, binary solvent manager, and a microprocessor-controlled multi-channel detector. Columns used included C18, C8, and amide types, each with a particle size of < 2 μm to ensure high-resolution separation.A C18 reverse-phase column (2.1 × 100 mm, 1.7 μm) was used for chromatographic separation. Solvent A (water with 0.1% formic acid) and solvent B (acetonitrile with 0.1% formic acid) made up the mobile phase. After 20 min of applying a linear gradient from 5% to 95% mobile phase B (methanol) against mobile phase A (water), re-equilibration took place. The injection volume was 5 µL, the column temperature was 40 °C, and the flow rate was kept at 0.5 mL/min. ESI (electrospray ionization) was used for detection in both positive and negative ion modes, with a mass scan range of m/z 100–1500.

The system operated with a mass accuracy of < 1 ppm and a resolving power greater than 40,000 full width at half maximum (FWHM), covering a mass range of 20–4000 m/z. Methanol was used as the mobile phase. Both positive and negative ESI modes were applied. ESI conditions included a capillary voltage of 2400 V, a desolvation temperature of 650 °C, and a source temperature of 150 °C^24^. The UPLC-MS analysis provided detailed data on molecular formula, molecular weight, retention time, m/z ratio, and relative abundance of each compound. Identification of compounds was achieved by matching the obtained spectra and chromatographic peaks with spectral libraries and database references.

Based on precise mass measurements and comparison with published literature, major ingredients such quercetin derivatives, oleuropein glucoside, and chemicals related to resveratrol were found. The diagnostic fragment ions described for these compounds were available and in agreement with earlier research, supporting their potential identification^25,26^. With little spectral support, certain low-abundance chemicals were found and showed no indications of contamination. Because of the potential for background contamination or carryover effects, these were categorized as preliminary identifications and were not highlighted in mechanistic explanations. Based on confidence level 2 were used for network pharmacology and docking analyses.

### FT-NMR analysis

Fourier-Transform Nuclear Magnetic Resonance (FT-NMR) analysis was performed using a Bruker Avance III spectrometer operating at 500 MHz, corresponding to a magnetic field strength of 117,000 gauss and a resonance frequency of 500.23 MHz for one-dimensional proton (^1H) NMR. The system was operated and processed using TopSpin 2.1 software. The polyherbal sample was dissolved in deuterated methanol (CD₃OD) with a deuteration degree of 99.8%. About 10 mg of polyherbal extract were dissolved in 0.6 mL of deuterated solvent to create NMR sample, which was then put into 5 mm NMR tube for examination. Tetramethylsilane (TMS) served as the internal reference standard. Chemical shifts were reported in parts per million (ppm), and the mean acquisition time for ^1H NMR spectra was 1.6384 s^27^.

### Extraction and characterization of polyphenols by UV visible spectroscopy

Quantitative Analysis of Phytonutrients and the extraction of polyphenols using the maceration methodfollowing the previously published paper^28^ for characterization.

UV-Visible spectrophotometry was employed to determine the maximum absorbance wavelength (λmax) of the polyphenolic extract. The polyphenolic concentrations ranging from 10 to 100 µg/mL were measured at room temperature (25 ± 2 °C) in quartz cuvettes with a 1 cm path length spanning a wavelength range of 200–800 nm. This technique is based on the principle that molecular compounds absorb ultraviolet or visible light, resulting in the excitation of electrons from their ground state to higher energy states. The interaction between light and matter produced a characteristic absorption spectrum, aiding in the qualitative evaluation of the extract’s bioactive constituents^29^.

### Antioxidant activity determination

One gram of the polyherbal powder was macerated in methanol, acetone, chloroform, ethyl acetate, and water separately and kept at 37 °C for 72 h. The mixtures were then filtered and concentrated at the same temperature to obtain a dark viscous extract, which was used for the DPPH radical scavenging assay^30^. The antioxidant activity of the polyherbal extracts was determined using the α,α-diphenyl-β-picrylhydrazyl (DPPH) method with slight modifications^30,31^. A 0.1 mM DPPH solution was prepared in methanol. Equal volumes of DPPH solution and polyherbal extract (ranging from 0.02 to 0.12 mg/mL) were mixed and incubated in the dark at 37 °C for 30 min. Absorbance was measured at 517 nm using a UV-Vis spectrophotometer. Ascorbic acid was used as the positive control. Solvent and DPPH without extract served as the blank (negative control). The percentage of radical scavenging activity (RSA) was calculated using the following formula:

$${\rm{RSA\% = [Optical\,\, Density\ (Blank) - Optical\,\, Density (Polyherbal\,\, Extract)/ Optical\,\, Density\,\, (Blank)] \times 100}}{\rm{.}}$$

### Determination of antimicrobial activity

The antimicrobial activity of the polyherbal extract was assessed against both bacterial and fungal pathogens using the disc diffusion method.

Bacterial Assay: Nutrient broth was inoculated aseptically with a loopful *of Staphylococcus aureus*,* Streptococcus mutans* and *Actinomyces viscosus*. The cultures were incubated overnight at 37 °C^32^. Mueller-Hinton agar was poured into sterile Petri dishes and allowed to solidify. The test organisms were evenly spread using sterile cotton swabs. Wells of 0.8 cm diameter were created using a sterile cork borer. Different concentrations (0.25, 0.375, and 0.5 mg) of the polyherbal extract were added to the wells. Tetracycline served as the positive control and negative control is solvent. Plates were incubated overnight at 37 °C for 24 h. Antibacterial efficacy was assessed by measuring the zone of inhibition around each well^33^.

Fungal Assay: *Candida albicans* was inoculated into potato dextrose agar (PDA) under sterile conditions and incubated overnight at 37 °C^34^. PDA was poured into sterile Petri dishes and allowed to solidify. C. albicans was evenly swabbed across the agar surface and incubated for 48 h. Wells were created in the agar using a cork borer, and varying concentrations of the polyherbal extract (0.25, 0.375, and 0.5 mg) were added. Fluconazole was used as the positive control and negative control is solvent. Plates were incubated at 28 °C for 48 h. Antifungal activity was determined based on the zone of growth inhibition surrounding each well^34,35^. The agar well diffusion method was used to assess antimicrobial activity; 6 mm diameter wells were punched in the agar, and 50 µL of the test sample was added to each well. Before plating, microbial inocula were prepared from fresh cultures and standardized to 0.5 McFarland turbidity (~ 1 × 10² CFU/mL) prior to plating; all experiments were carried out in technical triplicate.

According to CLSI (Clinical and Laboratory Standards Institute) guidelines, MIC (Minimum Inhibitory Concentration) was calculated using an agar well diffusion-based method. MIC is the lowest concentration of the extract that results in a clear zone of inhibition as compared to the solvent control.

### Anticancer activity determination

#### Cell line, culture conditions, and reagents

Human oral squamous carcinoma (OECM-1) cell lines were obtained from the National Centre for Cell Sciences (NCCS), Pune, India. The cells were cultured in Roswell Park Memorial Institute (RPMI) medium supplemented with 10% fetal bovine serum (FBS) and 1% penicillin-streptomycin. Cultures were maintained at 37 °C in a humidified atmosphere containing 5% CO₂. FBS was procured from Cistron Laboratories, and RPMI medium was obtained from HiMedia Laboratories, India. Phalloidin was purchased from Thermo Fisher Scientific. Trypsin, methylthiazolyl diphenyl-tetrazolium bromide (MTT), and dimethyl sulfoxide (DMSO) were procured from Sisco Research Laboratory Chemicals, Mumbai. All other analytical grade chemicals and reagents used in the experiments were sourced from Sigma-Aldrich, Mumbai, India.

#### In vitro anticancer activity: MTT assay

The anticancer activity of the polyherbal extract was evaluated using the MTT assay^36,37^. Before treatment, OECM-1 cells were sown in 96-well plates at a density of 5 × 10³ cells/well and given a full day to adhere. After that, the cells were exposed to the extract for 24 h. The polyherbal extract doses of 3, 25, 50 and 100 µg/mL were evaluated. Post-incubation, cells were washed with phosphate-buffered saline (PBS, pH 7.4), followed by the addition of 100 µl of MTT solution (5 mg/ml). The plates were incubated for 4 h, after which the formazan crystals formed were dissolved in 100 µl of DMSO. Absorbance was measured at 570 nm using a UV spectrophotometer, with DMSO serving as the negative blank and Diallyl disulfide (DADS) serving as the positive control.Cell viability was not significantly impacted by the final vehicle concentration, which did not surpass 0.1% (v/v). Cytotoxicity was additionally evaluated in Vero cells as a non-malignant control to assess selectivity. Three separate biological replicates (*n* = 3) were used for the assay, and each condition was examined in technical triplicate on separate days. Using nonlinear regression analysis of dose-response curves in GraphPad Prism, the half-maximal inhibitory concentration (IC₅₀) was determined; values are presented with 95% confidence intervals.

Cell viability was calculated using the formula:

$${\rm{\% Cell}}{\mkern 1mu} {\mkern 1mu} {\rm{Viability = }}\left( {{{\rm{A}}_{{\rm{570}}}}{\mkern 1mu} {\mkern 1mu} {\rm{of}}{\mkern 1mu} {\mkern 1mu} {\rm{treated}}{\mkern 1mu} {\mkern 1mu} {\rm{cells/}}{{\rm{A}}_{{\rm{570}}}}{\mkern 1mu} {\mkern 1mu} {\mkern 1mu} {\mkern 1mu} {\rm{of}}{\mkern 1mu} {\mkern 1mu} \,\,{\rm{control}}{\mkern 1mu} {\mkern 1mu}\,\, {\rm{cells}}} \right){\rm{ \times 100}}{\rm{.}}$$

Graphs were plotted with the percentage of cell survival on the Y-axis and the concentration of the test sample on the X-axis. IC₅₀ values (concentration required for 50% inhibition of cell viability) were determined.

#### Phalloidin staining for morphological assessment

To assess the morphological changes induced by the polyherbal extract, phalloidin staining was performed. OECM-1 cells were treated with various concentrations of the extract for 24 h. The cells were then fixed with paraformaldehyde, permeabilized with Triton X-100, and blocked using 5% bovine serum albumin (BSA). After incubation with phalloidin for 40 min, cellular morphology was visualized using fluorescence microscopy at 60× magnification^38^.

### Determination of anti-inflammatory activities

The anti-inflammatory activity of the samples was evaluated by quantifying the levels of pro- and anti-inflammatory cytokines—IL-10, IL-1β, IL-6, TNF-α, and TGF-β—using commercially available human ELISA (Enzyme Linked Immuno Sorbent Assay) kits (Boster Biological Technology, Pleasanton, USA). The kits used included: IL-6 PicoKine™ ELISA Kit (Catalog No. EK0410), IL-10 ELISA Kit (EK0416), TNF-α ELISA Kit (EK0525), IL-1β ELISA Kit (EK0394), and TGF-β ELISA Kit (EK0513). Following the manufacturer’s protocol, 100 µL of sample diluent, human standard, or test sample was added to each well and incubated at room temperature for 120 min. After removing the contents, 100 µL of 1× biotinylated antibody specific to each cytokine was added and incubated for 90 min at room temperature. Wells were then washed with buffered saline, followed by the addition of 100 µL of Avidin–Peroxidase–Biotin complex and incubation for 40 min at room temperature. Subsequently, 90 µL of color-enhancing substrate was added to each well and incubated in the dark for 30 min. The reaction was stopped with 100 µL of stop solution, resulting in a color change to yellow. Absorbance was measured at 450 nm using a microplate reader. After stimulating cells for 24 h with lipopolysaccharide (LPS, 1 µg/mL), cytokine levels (IL-1β, IL-6, TNF-α, IL-10, and TGF-β) were measured from cell culture supernatants using ELISA. Cell viability was evaluated using the MTT test in parallel at the same concentrations and exposure times to make sure that cytokine modulation was not a result of cytotoxicity. Only concentrations that maintained ≥ 80% cell viability were used to interpret cytokine data, suggesting that observed alterations were due to immunomodulatory effects rather than cell death. The positive inflammatory control was cells activated with lipopolysaccharide (LPS, 1 µg/mL), while the negative control was untreated cells. By contrasting LPS-treated cells with and without the samples, the impact of the samples on cytokine production was assessed. The concentration of each cytokine was calculated based on the standard curve.

### Statistical analysis

GraphPad Prism v9.0 was used for statistical analysis. Tukey’s post-hoc test and one-way ANOVA were used to compare groups. When appropriate, precise p-values and 95% confidence ranges are presented, with statistically significance set at *p* < 0.01. All experiments were conducted in triplicates, and each test was performed using three independent biological replicates (*n* = 3) and n representing independent biological replicates. The results were expressed as the arithmetic mean ± standard deviation (SD) and when necessary, post-hoc comparisons were carried out. Statistical significance of the results was assessed using one-way analysis of variance (ANOVA) was used to compare groups statistically and differences were deemed statistically significant at *p* < 0.01 through Fisher’s F-test, using suitable online statistical tools.

### Determination of polyphenols by network pharmacology

#### Structure and potential targets of polyphenols

Based on the availability of canonical SMILES and dependable detection (signal-to-noise ratio ≥ 10), Polyphenolic compounds identified from the UPLC-MS analysis were subjected to structural and target analysis through network pharmacology approaches.Only substances with clear molecular formulae were kept for additional examination. Their 2D and 3D SDF structures, along with canonical SMILES representations, were retrieved from the PubChem database (https://pubchem.ncbi.nlm.nih.gov/) to facilitate ligand preparation. Using the term “oral carcinoma,” to identify oral carcinoma genes in the database GeneCards (https://www.genecards.org/). Relevance scores were used to identify high-confidence genes, and the UniProt database was used to standardize gene symbols and UniProt IDs. To identify putative human protein targets of each compound Swiss Target Prediction web tool (http://www.swisstargetprediction.ch/), choosing the target species to be Homo sapiens. Only targets with high predicted probability scores were kept for each compound. A non-redundant target list for each of the 13 compounds was produced by combining the gathered data and eliminating duplicates(accessed January–February 2025). Using the relevance score levels suggested by each database, targets with Z-score probability ≥ 0.1 (Swiss Target Prediction) were chosen.This integrative analysis enabled the mapping of polyphenols to their respective protein targets and potential roles in oral carcinoma therapy.

#### Network analysis of protein–protein interactions (PPI)

Venny 2.1.0 was used to determine the intersection between compound-predicted targets and genes linked to oral carcinoma (https://bioinfogp.cnb.csic.es/tools/venny/). In order to combat oral carcinoma, the phenolic chemicals were thought to be effective therapeutic targets for the overlapping genes. These intersecting targets were then used for enrichment analysis and network development. To create a PPI network, the overlapping gene list was submitted to the STRING database (https://string-db.org/) using a medium confidence level of 0.4. Cytoscape (version 3.10.4) was used to visualize and perform a topological analysis on the interaction data. To find hub genes that potentially have regulatory roles in the network, network characteristics like degree centrality, betweenness centrality, and clustering coefficient were computed using Cytoscape’s Network Analyzer plug in. The nodes that ranked highest on centrality indicators were identified as important hub targets for additional research.

The Kyoto Encyclopedia of Genes and Genomes (KEGG) pathway enrichment and Gene Ontology (GO) functional enrichment, including Biological Process (BP), Cellular Component (CC), and Molecular Function (MF) categories, were carried out in R (version 4.5.2) using the cluster Profiler, org.Hs.eg.db, and enrich plot packages; a corrected p-value (Benjamini–Hochberg method) < 0.05 was deemed statistically significant. Enrichment plots and bubble diagrams were created to show the top enriched GO terms and KEGG pathways related to oral carcinoma treatment. Node size and colour in the network visualization were configured to reflect the degree of connectivity (edge count), with a gradient mapping of “low values to small sizes” and “low values to bright colours” to highlight central nodes in the network^39–41^. This approach allowed visualization of the complex interactions among polyphenolic compounds, protein targets, and signalling pathways involved in oral carcinogenesis.

#### Docking investigations and homology modelling

Using molecular docking, the chosen hub proteins were employed as possible therapeutic targets. Target protein 3D crystal structures were acquired from the Protein Data Bank (PDB) (https://www.rcsb.org/), with a preference for high resolution (< 2.5 Å) structures. PyRxv0.8 was used to prepare protein structures by eliminating heteroatoms, water molecules, and co-crystallized ligands (https://pyrx.sourceforge.io/). After geometry optimization and energy minimization using Open Babel, ligand files (SDF) were transformed into PDBQT format. AutoDock Vina was used to run docking simulations, and binding affinities (kcal/mol) were noted. PyMOL and BIOVIA Discovery Studio v3.7.1 were used to show molecular interactions such hydrophobic contacts and hydrogen bonding^39^. Molecular docking studies were conducted to further investigate the binding affinity of selected polyphenols toward key therapeutic targets associated with oral carcinoma. Five primary target proteins were chosen based on the PPI and pathway analyses with PDB IDswere as follows: AKT1 (AKT serine/threonine kinase 1) (4EJN), TP53 (Tumor protein p53) (1TUP), TNF-α (Tumor necrosis factor) (1TNF), EGFR (Epidermal growth factor receptor) (1M17) and STAT3 (Signal transducer and activator of transcription 3) (6NUQ). Docking grids were defined around the active sites and binding affinities were evaluated using Vina’s scoring function. By re-docking co-crystallized ligands, docking reliability was evaluated and all docking results are interpreted as predictive and hypothesis-generating. The suggested technique is an experimental and systems pharmacology based framework, incorporating phytochemical profiling, in vitro bioassays, and network pharmacology analysis, rather than a mathematical or theoretical modeling investigation (Supplementary Fig. 23).

## Results and discussion

### Phytochemical profile of the polyherbal extracts

The phytochemical analysis of the polyherbal mixture revealed the presence of several key bioactive constituents, including carbohydrates, proteins, amino acids, tannoids, alkaloids, saponins, flavonoids, glycosides, and polyphenols across various solvent extracts. However, carotenoids and fatty acids were consistently absent in all the extracts tested. Among the different solvents, the methanol extract exhibited the highest concentration and diversity of phytochemicals, followed by the chloroform and aqueous extracts (Supplementary Table 1). This suggests that methanol is a more efficient solvent for extracting a broad range of phytoconstituents from the polyherbal formulation. These phytochemicals are recognized for their diverse biological activities. They contribute significantly to health promotion by combating oxidative stress and inflammation and may play a role in the prevention of chronic diseases such as cancer, cardiovascular disorders, arthritis, neurodegeneration, and immune dysfunction^42^.

### Phytonutrient profiling by GC-MS analysis

Gas chromatography-mass spectrometry (GC-MS) was performed on the methanolic extract of the polyherbal formulation to determine its phytonutrient composition. The chromatographic spectra, represented in Supplementary Fig. 1 & Supplementary Fig. 2, revealed a total of 20 distinct phytochemical constituents. Among these, the most abundant compounds based on the peak area (hit %) included Desulphosinigrin (32.24%), W-18 (30.46%), 3-O-Methyl-d-glucose (28.04%), Phytol (22.44%), (13Z,10Z,7Z)-Hexadeca-13,10,7-trienal (10.53%), Neophytadiene (7.82%), and Undecane (14.81%). These bioactive components may contribute significantly to the polyherbal formulation’s therapeutic properties. The total ion chromatogram (TIC) enabled the identification of each compound through comparison with reference spectra, revealing retention time, molecular formula, molecular mass, and relative abundance (Table 1). Each of these phytochemicals has been previously reported in the literature with proven pharmacological properties, including anti-inflammatory, antioxidant, antimicrobial, cytotoxic, antidiabetic, immunomodulatory, and anticancer activities. A few notable examples include Undecane and Tridecane, which are known for antimicrobial, antiviral, and cytotoxic activities^43,44^. Desulphosinigrin has been reported in Balarishta and exhibits anti-rheumatic, antioxidant, and urease-inhibitory properties^45^. Phytol is widely recognized for its anti-inflammatory, anticarcinogenic, and antimalarial activities^46^. W-18 exhibits potent neuroprotective activity with potential application in Alzheimer’s therapy^47^. These results demonstrate the diverse bioactivity of the polyherbal extract and provide a scientific rationale for its traditional use in managing various ailments.

**Table 1:** Compounds of the polyherbal extract identified by GC-MS.

S.No	Retention mins	Phytonutrients	Chemical formula	Molecular mass (gm/mol)	Hit region%
1	6.241	Undecane	C_11_H_24_	156.31	7.56
2	6.241	Tridecane	C_13_H_28_	184.37	7.56
3	6.241	Decane	C₁₀H₂₂	142.29	7.56
4	18.798	Desulphosinigrin	C_10_H_17_NO_6_S	279.31	17.09
5	18.798	L-Glucose	C_6_H_12_O_6_	180.16	17.09
6	18.798	Stevioside	C_38_H_60_O_18_	804.87	17.09
7	19.459	3-O-Methyl d-glucose	C_7_H_14_O_6_	194.18	37.03
8	19.459	3-Methylmannoside	C_7_H_14_O_6_	194.18	37.03
9	19.459	Trehalose	C_12_H_22_O_11_	342.29	37.03
10	23.643	Neophytadiene	C_20_H_38_	278.5	13.94
11	23.643	15, 11, 7, 3-Tetraalkyl-2,1-cetene-1-ol	C_20_H_39_O_2_	311.52	13.94
12	23.643	9-Eicosyne	C_20_H_38_	278.5	13.94
13	30.406	(13Z, 10Z, 7Z) Hexadeca 13,10,7- trienal	C_16_H_25_O_2_	250.37	12.58
14	30.406	15,12,9-Octalinolenic acid, (3Z)-	C_18_H_30_O	278.42	12.58
15	30.406	Methyl 8,11,14- heptadecatrienoate	C_18_H_30_O_2_	278.4	12.58
16	30.618	Phytol	C_20_H_40_O	296.53	6.23
17	30.618	9-Octadecene, 1,1-dimethoxy-, (Z)-	C_20_H_40_O	312.53	6.23
18	30.618	cis-1,2-Cyclododecanediol	C_12_H_24_O_2_	200.32	6.23
19	42.739	W-18	C_19_H_20_ClN_3_O_4_S	421.9	5.58
20	42.739	Hexasiloxane, 1,1,3,3,5,5,7,7,9,9,11,11- dodecamethyl-	C_12_H_36_O_5_Si_6_	428.9	5.58

### Phytochemical compounds identified by UPLC analysis

Ultra-Performance Liquid Chromatography (UPLC) analysis of the methanolic extract of the polyherbal formulation revealed the presence of 22 phytochemical compounds, as shown in Supplementary Fig. 3&4. These compounds were identified based on their retention time, mass-to-charge ratio (m/z), molecular weight, molecular formula, and by comparison with reported literature data. A wide range of phytochemical classes was detected, including phenols, terpenoids, flavonoids, alkaloids, saponins, glycosides, coumarins, and steroids, demonstrating the rich chemical diversity of the polyherbal mixture (Table 2). These phytocompounds have numerous biological significances such as Quercetin derivatives, such as quercetin O-rhamnoside-O-hexoside and quercetin pentoside, are potent antioxidants and acetylcholinesterase inhibitors with therapeutic potential against oxidative stress-related diseases^26^. Resveratrol dimer, found in red grapes, peanuts, and mulberries, is well known for its anti-inflammatory, anticancer, antimicrobial, cardioprotective, immunomodulatory, radioprotective, and neuroprotective activities^48^. Sinapic acid, a common polyphenol in citrus fruits, shows a wide range of bioactivities, including anti-carcinogenic, antihyperglycemic, anti-inflammatory, antioxidant, antibacterial, and anxiolytic effects^49^. Myricetin, a naturally occurring bioflavonoid, has broad pharmacological potential due to its anticancer, antidiabetic, antioxidant, anti-inflammatory, and neuroprotective activities^50^. Quercetin is a phenolic compound possesses excellent free radical scavenging ability^51^.

Previous UPLC–MS based studies have reported many polyphenolic compound identification based on m/z values relevant for species distinction. The separation of Asian species in PCA (Principal Component Analysis) was greatly aided by feruloyl tyramine 4-O-hexoside (m/z 476) (Geng et al., 2017). MS/MS fragmentation was used to confirm Musa acuminata markers like quercetin O-rhamnoside-O-hexoside (m/z 609)^26^. Oleuropein glucoside (m/z 701), isorhamnetin-3-O-glucoside (m/z 475), resveratrol dimer (m/z 453), myricetin (m/z 274), sinapic acid (m/z 208), syringaresinol (m/z 387), catechol dimer (m/z 218), quercetin pentoside (m/z 433), quercetin [H₂O]+ (m/z 437), and puerarin (m/z 415) have all been detected in different plant matrices using UPLC-MS^25,51,55^.

**Table 2:** Compounds identified by UPLC in methanol extract of polyherbal mixture.

Compound	Peak	m/z	%	Mol. formula	Type of compound	Mol.Wt (gm·mol − 1)
Protopanaxatriol (PPT) aglycone types of ginsenosides	6.31	475	100	C_30_H_52_O_4_	Saponin	476.732
Feruloyl tyramine 4-O-hexoside	6.31	476	18	C_24_H_29_NO_9_	Phenolic Glycoside	475.5
Quercetin O-rhamnoside-O hexoside	6.75	609	100	C_27_H_30_O_16_	Flavonoid	610.5
Deoxy- ecdysone	6.75	610	19	C_27_H_44_O_5_	Steroid	448.64
Oleuropein glucoside	6.9	701	100	C_25_H_32_O_13_	Phenol	540.518
17-demethoxy-reblastatin	6.9	679	37	C_28_H_42_N_2_O_8_	Glucoside	557.28
Resveratrol dimer	7.38	453	60	C_28_H_22_O_6_	Phenol	454.47
Isorhamnetin-3-O-glucoside	7.38	475	30	C_22_H_22_O_12_	Flavonoid and Glycoside	478.4
Sinapic acid	8.36	208	100	C_11_H_12_O_5_	Phenol	224.21
Isoprene-derived organosulfates	9	212	100	C_5_H_8_	Terpenoid	68.12
Vitamin E – Tocopherol	9	213	10	C_28_H_48_O_2_	Phenol	430.706
4′-O-acetyl-3-C-methyl-rhodosamine	9	214	8	C_8_H_10_O_2_	Coumarins	138.16
Senkirkine	9	168	9	C_19_H_27_NO_6_	Alkaloid	365.4
Nodakenin	10.64	409	100	C_28_H_32_O_13_	Coumarins	408.4
Syringaresinol	10.64	387	50	C_22_H_26_O_8_	Phenol	418.437
Tri Hydroxylated phytoecdysteroid	10.64	432	33	C_27_H_44_O	Terpenoid	480.6
Catechol dimer (Ox1)	10.64	218	11	C_6_H_6_O_2_	Flavonoid	110.112
Quercetin pentoside	10.64	433	5	C_25_H_26_O_15_	Phenolic Flavonoid	566.5
Myricetin	10.96	274	100	C_15_H_10_O_8_	Phenolic Flavonoid	318.24
Quercetin [H2O]+	11.57	437	100	C_15_H_10_O_7_	Phenolic Flavonoid	302.235
Simazine	11.57	174	8	C_7_H_12_C_l_N_5_	Terpenoid	201.66
Puerarin	11.57	415	20	C_21_H_20_O	Flavonoid	416.38

Polyphenols are widely associated with anticancer activity. Due to their polar and thermolabile nature, phenolic compounds are preferentially detected by UPLC-based techniques rather than GC–MS which mainly identifies volatile and non-polar constituents^52^. Accordingly, compound selection for in silico analyses in this study was based on UPLC-identified polyphenolic constituents.

### FT-NMR

NMR spectrum of methanol extract of the polyherbal formulation is shown in Supplementary Fig. 5. The NMR peaks corresponding to the chemical shift indicated that alkyl (0.7-2ppm), allylic H (1.6-2ppm), ketone and benzylic (2.1-2.1.8ppm), acetylenic (2.5–3.5.1ppm), alcohol (3.4–4.4), alkyl halide (3.2-3.2.9ppm), ether (3.4-3.4.9ppm), amine (4.5–6.5.7ppm), vinylic (4.6–5.6.6ppm), phenolic (4.5–6.5.9ppm), alkenes (5.5-6ppm), amide (5.5–8.5.5ppm), aromatic (6.1–8.1.5ppm) groups are present on 1 H NMR spectroscopy. The chemical frequency of values between 6.80 and 9.22 parts per million revealed the appearance of aromatic compounds in a study on 1 H NMR analysis of non-thermal processing of orange juice for the ozone treatment (EG et al., 2016). The range is between 5.9 and 5.1 ppm confirming the presence of vinyl protons (-CH=CH2) in a study on 1 H magnetic resonance spectrometer exhibited the clear cut nature of the biopolymers. This could open routes of novel polythene-based biopolymers^53^. Range between 7.94 and 7.06 ppm showed the presence of aromatic protons signal in ortho and meta positions in a study on 1 H NMR spectrum analysis of cannabinoids. The chemical shift at 3.97ppm revealed the presence of ethoxy group in meta position. The chemical shift in the value between 7.78 and 7.73 parts per million showed the appearance of benzene group with amide in ortho position. The chemical shift at 7.40 ppm and 7.47ppm revealed the presence of benzene group with amide in meta position and para position respectively^54^.

The chemical shift presents at 6.36 ppm displayed the appearance of benzenolic constituents of gamma-tocopherol. The range between 6.8 and 6.6 ppm showed the presence of dodecyl gallate. The chemical shift present at 7.2 ppm showed the presence of hydroxyl tyrosol acetate. This data was based on the 1 H NMR spectrum of the virgin flaxseed oil that showed good antioxidant activity^55^.

### Extraction and characterization of polyphenols by UV visible spectroscopy

Quantitative Analysis of Phytonutrients showed higher phenolic concentration than other constituents including flavonoids and tannin. UPLC analysis revealed that 13 of the 25 detected compounds were polyphenols. Based on quantitative and UPLC analysis, the polyphenols was extracted by maceration which was 476.63 µg GAE/mL^28^.

The determination of PPF from methanolic leaf extract was carried out using the double beam spectrophotometer. The UV absorbance of PPF from methanolic leaf extract has main peak at 272 nm. The results of the UV analysis of the PPF suggested that phenols were present in the methanolic leaf extract (Supplementary Fig. 6). In previous research, the UV visible spectroscopy of Spiraea japonica methanolic leaf extract confirms the phenol peak present at 272 nm^56^. Successful extraction and phytochemical integrity were confirmed by the observed absorption peaks and identified phytochemical ingredients, which matched previously reported bioactive chemicals in medicinal plants^57^.

### Antioxidant activity of the polyherbal extracts

The antioxidant potential of the polyherbal extracts was evaluated using DPPH free radical scavenging assay. Among all extracts tested, the methanol and aqueous extracts demonstrated the most potent scavenging activities with IC_50_ values of 108 ± 0.17 µg/mL and 112 ± 0.22 µg/mL, respectively (Supplementary Fig. 7). The acetone and chloroform extracts exhibited moderate antioxidant activity with IC_50_ values of 140 ± 0.16 µg/mL and 171 ± 0.24 µg/mL, respectively. The ethyl acetate extract showed the weakest activity, with an IC_50_ value exceeding 250 µg/mL. For comparison, the standard antioxidant vitamin E exhibited a significantly lower IC_50_ value of 87 ± 0.10 µg/mL, indicating its higher scavenging efficiency. The radical scavenging activity of the extracts was dose-dependent, increasing with higher concentrations of polyherbal extract. Among the samples, the methanolic extract displayed the strongest antioxidant effect and was therefore selected for subsequent experiments. Polyherbal formulations are particularly advantageous due to their synergistic effectsthan individual effects. For instance, polyherbal mixtures comprising *Khaya senegalensis*,* Camellia sinensis*,* Phyllanthus species*,* Zingiber officinale* and *Nauclea latifolia* exhibited significantly stronger antioxidant activity compared to the individual plant extracts, as demonstrated in DPPH assays using vitamin C as standard^58^. These results support the growing recognition of polyherbal mixtures as effective sources of natural antioxidants. The extract showed moderate efficacy in comparison to the standard antioxidant and a concentration-dependent increase in antioxidant activity as compared to the untreated control.

### Antimicrobial activity of the polyherbal extracts

The antimicrobial potential of the polyherbal mixture was assessed using the agar well diffusion method against oral cavity pathogens, including *Streptococcus mutans*,* Staphylococcus aureus*,* Actinomyces viscosus*, and *Candida albicans*. Among the various solvent extracts tested, the methanolic extract demonstrated the highest antimicrobial activity, followed by acetone and ethyl acetate extracts. Notably, the methanol extract exhibited significant antibacterial activity against *S. aureus*, with a maximum inhibition zone of 21.33 ± 0.57 mm at 500 µg/mL, exceeding the efficacy of the standard antibiotic tetracycline (20.33 ± 0.57 mm). *S. mutans*, however, showed the least susceptibility to all tested extracts. The antifungal activity was also noteworthy, with maximum inhibition against *C. albicans* (14.00 ± 1.00 mm) at 500 µg/mL, surpassing the standard fluconazole (12.33 ± 0.57 mm). In general, the antimicrobial efficacy followed the order: *S. aureus > A. viscosus > C. albicans > S. mutans*, and a dose-dependent increase in the zone of inhibition was observed for all extracts (Supplementary Fig. 8 & Supplementary Table 2). For every oral pathogen studied, the lowest dosage that produced a distinct inhibitory zone above the solvent control was 250 µg/well, which was regarded as the apparent MIC. Concentration-dependent inhibition was confirmed by bigger inhibition zones at higher doses. The species with the lowest inferred MIC were Streptococcus mutans, Actinomyces viscosus, Candida albicans, and Staphylococcus aureus (Supplementary Fig. 9). In previous work, the formulations containing *Solanum xanthocarpum* (kantakari) and mastic have shown substantial inhibitory effects against *S. mutans*,* Lactobacillus* and *A. viscosus*, suggesting their potential application in oral hygiene products such as dentifrices and mucosal cleansers^59^. Collectively, these findings underscore the therapeutic promise of polyherbal mixtures as effective, natural antimicrobial agents especially in the context of oral health management. The extract’s antimicrobial activity was considerably higher than that of the negative control but lower than that of the conventional antibiotic, suggesting a moderate level of inhibitory capability.

### Anticancer activity

**Fig. 1:**
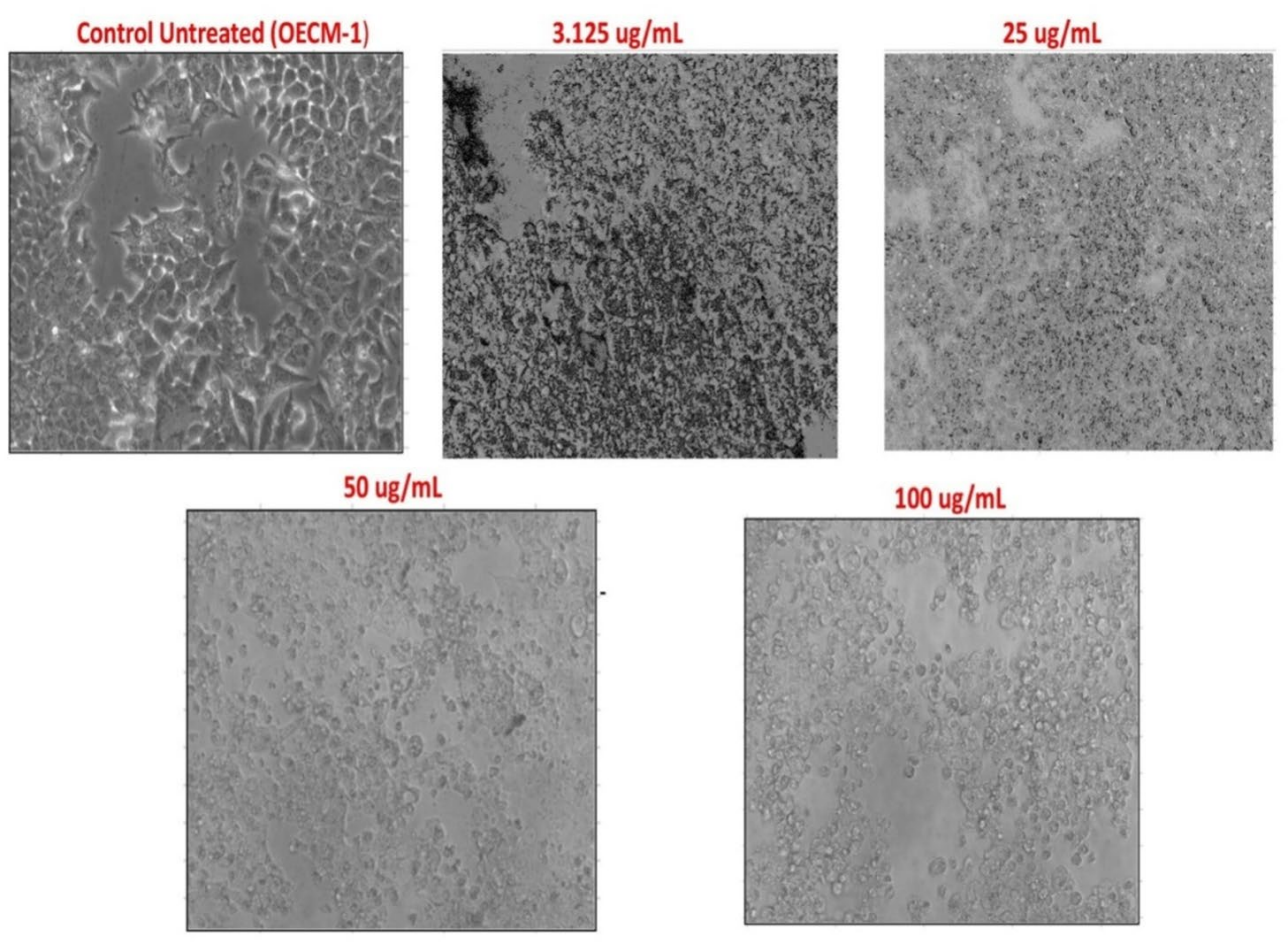
Anticancer activity of polyphenolic extract against OECM – 1 cell line.

**Fig. 2:**
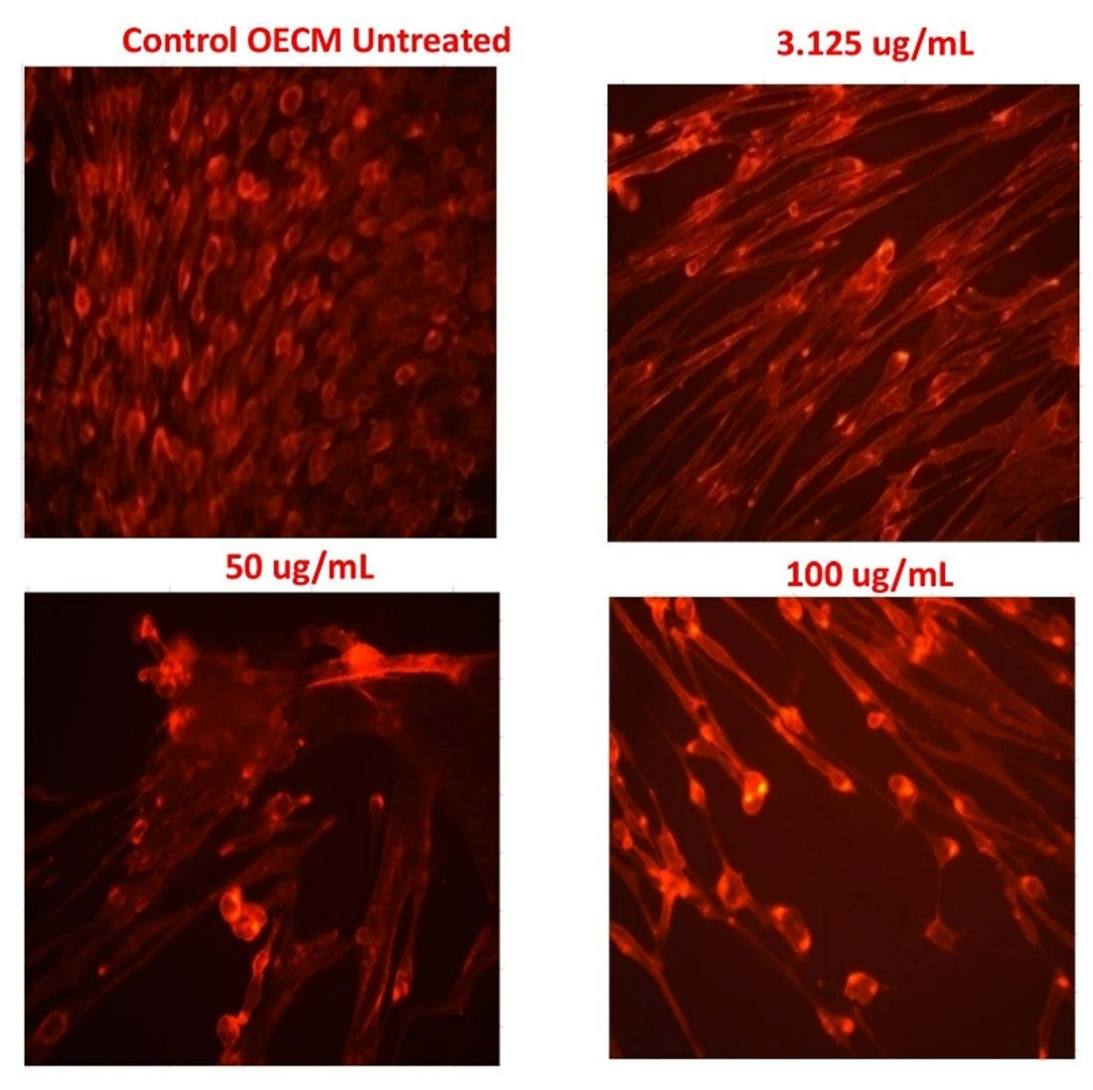
Anticancer activity against OECM – 1 cell line with phalloidin staining.

The anticancer potential of the polyphenolic extract was evaluated using the MTT assay on the OECM-1 oral carcinoma cell line. At a concentration of 0.1 mg/mL, the positive control diallyl disulfide exhibited 41.26% cell viability, while the polyphenolic extract showed 42.02% cell viability, indicating comparable cytotoxic activity. The extract demonstrated potent dose-dependent antiproliferative activity, with an IC₅₀ value of 18.86 µg/mL, suggesting strong therapeutic potential (Figs. 1 and 2, Supplementary Fig. 9).The lower cytotoxic effect observed in Vero cells with an IC₅₀ value of 54.34 µg/mL, compared to OECM-1 cells indicates preferential anticancer activity (Supplementary Figs. 11 and 12).

Phenolic compounds, such as pterostilbene, have been reported to possess strong antioxidant and anticancer properties, acting through the reduction of reactive oxygen species (ROS) and inducing apoptosis in cancerous cells^60^. In a similar study, the MTT assay of *Rheum ribes* showed an IC₅₀ of 250 µg/mL against OECM-1 cells, emphasizing the superior potency of the polyphenolic extract reported in this study^61^. Although the effect was not as strong as that of the conventional anticancer medication, cell viability was considerably decreased in extract-treated cells in comparison to untreated controls in a dose-dependent manner.

### Anti-inflammatory activities

**Fig. 3:**
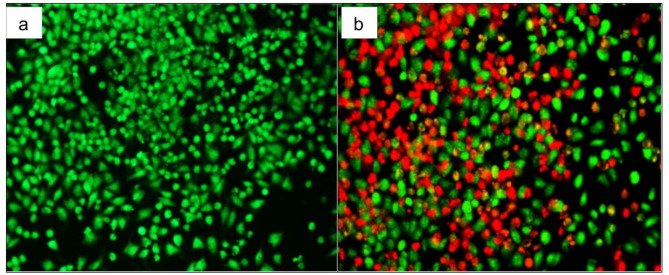
OECM-1 cell line (**a**) Control OECM-1 (**b**) LPS Induced cells- Dead cells are indicated by red, and necrosis is indicated by yellow.

**Fig. 4:**
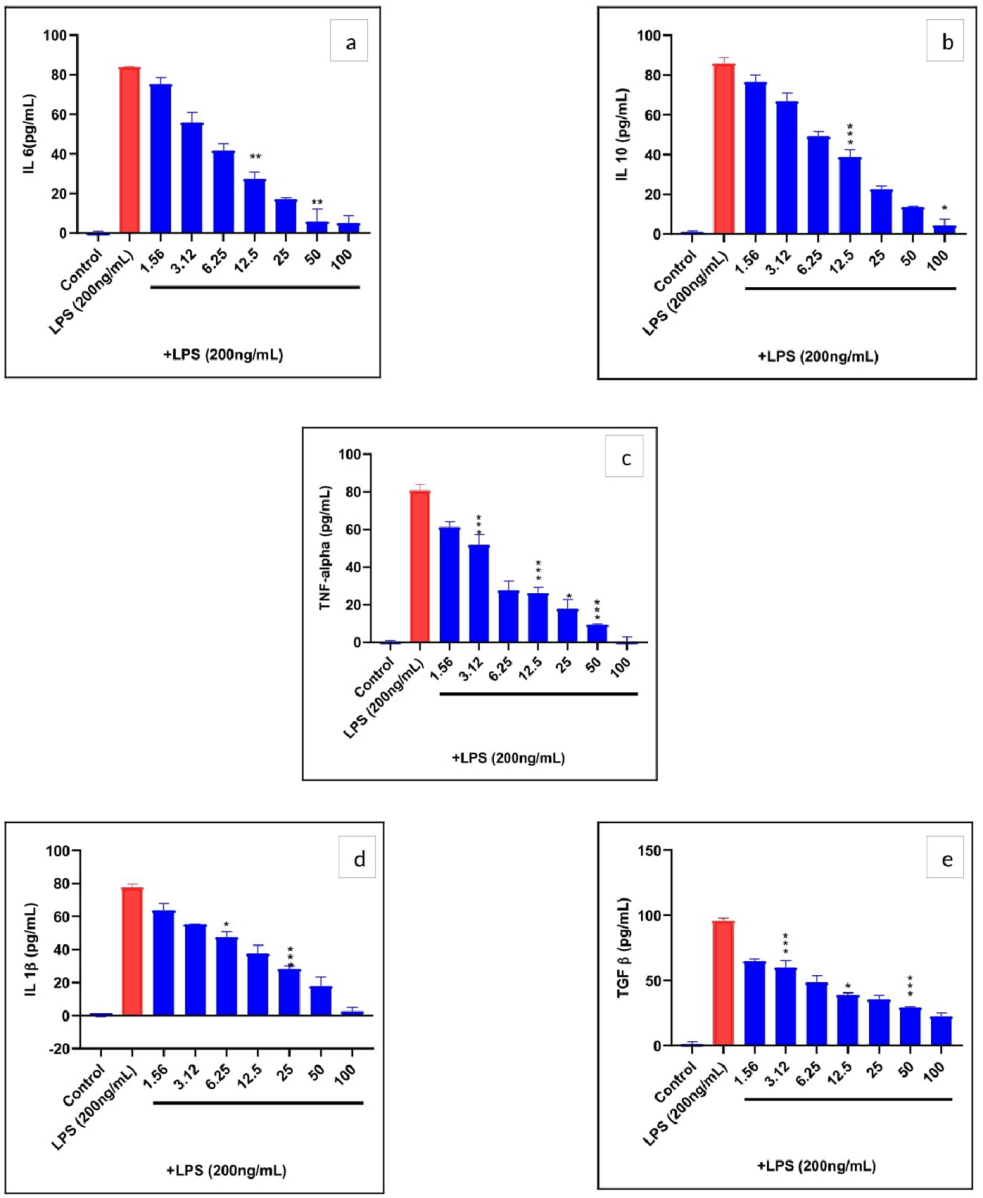
Inflammatory cytokines (IL6, IL10, TGF beta, IL 1 beta and TNF alpha) (**a**) IL6 (**b**) IL10 (**c**) TNF Alpha (**d**) IL1 Beta (**e**) TGF Beta.

The anti-inflammatory efficacy of the polyphenolic extract was assessed through cytokine profiling of LPS-induced OECM-1 cells, with particular focus on inflammatory markers: IL-6, IL-10, TNF-α, IL-1β, and TGF-β. The extract significantly downregulated the expression of all five cytokines in a dose-dependent manner. The IC₅₀ values for each cytokine were as follows: TNF-α: 3.02 µg/mL, IL-6: 4.10 µg/mL, IL-1β: 4.53 µg/mL, IL-10: 6.14 µg/mL, TGF-β: 6.15 µg/mL. TNF-α was the most significantly suppressed cytokine, indicating a strong anti-inflammatory profile (Figs.3 and 4). When compared to the LPS control, treatment with the extract dramatically decreased the levels of pro-inflammatory cytokines caused by LPS, while modulation of regulatory cytokines indicated a context-dependent anti-inflammatory impact.

In earlier studies, the biological interpretation for the polyphenolic extract’s apparent modification of cytokine expression. While anti-inflammatory and anti-tumorigenic effects are consistent with significant suppression of conventional pro-inflammatory cytokines like TNF-α, IL-1β, and IL-6, concomitant regulation of regulatory cytokines like TGF-β and IL-10. While TGF-β and IL-10 are known for their immunoregulatory functions, there is growing evidence that both cytokines support carcinoma cell survival, tumor immune evasion, and the suppression of effective anti-tumor immune responses within the tumor microenvironment^62,63^. Specifically, in oral squamous cell carcinoma, abnormal TGF-β signaling has been closely linked to the epithelial–mesenchymal transition, tumor development, and metastasis^64^. Therefore, rather than generic immunosuppression, the downregulation of IL-10 and TGF-β seen in this study is more likely to be linked to disruption of tumor-promoting immunoregulatory mechanisms^65^.

The current study used OECM-1 oral squamous cell carcinoma cells to assure disease relevance, despite the fact that macrophage or monocyte cell lines are frequently used as standard inflammatory models. Through TLR4 (Toll like receptor 4) -mediated LPS responsiveness and cytokine release, oral epithelial carcinoma cells actively contribute to the development of the tumor-associated inflammatory environment^66^. In response to LPS stimulation, oral epithelium and OSCC cells have been shown to display functional TLR4 signaling machinery and to modulate both pro-inflammatory cytokines (IL-1β, IL-6, TNF-α) and regulatory cytokines (IL-10, TGF-β). Consequently, compared to immune-cell-only inflammatory models, OECM-1 cells enable assessment of tumor-intrinsic cytokine modulation, which is more directly related to the development of oral carcinoma. The polyphenolic extract from the polyherbal mixture exhibits potent anticancer and anti-inflammatory properties, may reduce cancer cell viability and inflammatory cytokine expression. These findings support its potential use as a bioactive therapeutic agent in the management of oral carcinoma and inflammation-associated disorders.

### Network pharmacology of polyphenols

#### Potential targets of polyphenols

**Fig. 5:**
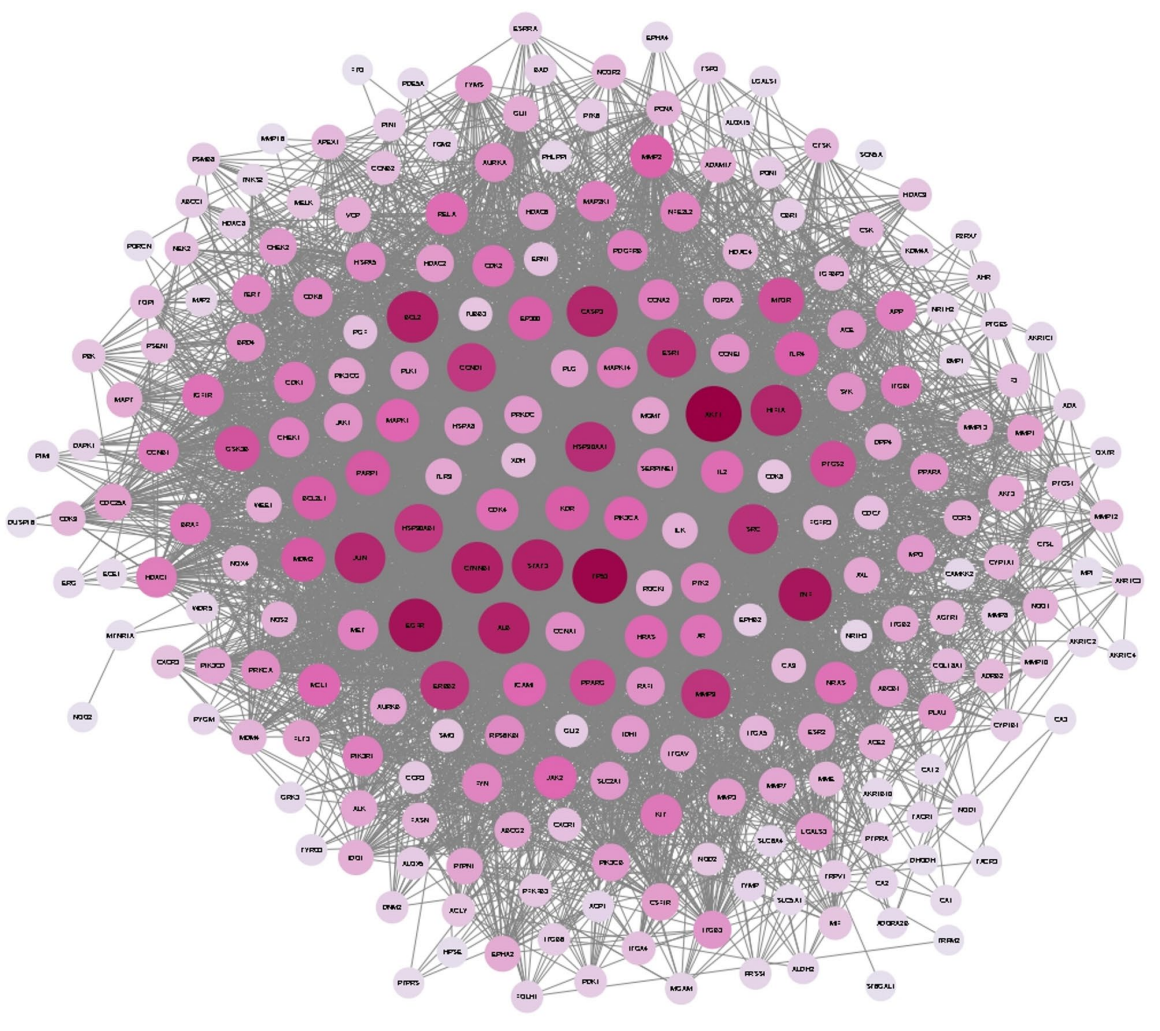
String analysis of protein- protein interaction network.

A total of 3,266 potential targets for selected polyphenolic compounds were identified through SwissTargetPrediction. The polyphenols evaluated included feruloyl tyramine 4-O-hexoside, quercetin O-rhamnoside-O-hexoside, oleuropein glucoside, resveratrol dimer, isorhamnetin-3-O-glucoside, sinapic acid, vitamin E (tocopherol), syringaresinol, myricetin, quercetin [H₂O]+, catechol, quercetin pentoside, and puerarin. After eliminating duplicate entries and irrelevant hits, a refined list of 277 unique target genes was compiled. Upon comparison with oral carcinoma-associated genes (2216 genes), 251 overlapping targets were identified (Supplementary Fig. 13). These shared targets suggest a potential molecular mechanism through which polyphenols may exert therapeutic effects in oral carcinoma. Further protein–protein interaction (PPI) analysis was conducted using the STRING database, leading to the construction of a PPI network involving the 251 overlapping protein targets (Fig. 5).

Polyphenols are known to interfere with the initiation, promotion, and progression phases of carcinogenesis. By inducing apoptosis and inhibiting cancer cell proliferation, dietary polyphenols contribute significantly to oral carcinoma prevention^67^. Prior in silico investigations, such as those focused on myricetin, have demonstrated its anti-cancer potential. Notably, 22 shared target genes were identified between myricetin and oral cancer, implicating biological pathways such as chemical carcinogenesis–reactive oxygen species (ROS), PI3K-Akt signaling, microRNA regulation, and the Ras signaling pathway. Among the key hub genes associated with the top 10 enriched pathways were AKT1 and EGFR. The therapeutic potential of myricetin in oral carcinoma appears to be strongly associated with EGFR-mediated signaling, particularly in conjunction with AKT1 activation^68^.

#### Polyphenols and oral carcinoma: overlapping targets and enrichment analysis

A total of 251 overlapping targets between polyphenols and oral carcinoma were analyzed using GO and KEGG enrichment. GO enrichment analysis revealed significant biological processes including responses to epithelial cell proliferation (GO:0050673), xenobiotic stimulus (GO:0009410), cellular response (GO:0035924), cellular component and molecular function(Supplementary Fig. 14, Supplementary Table 3).

KEGG pathway analysis identified 20 pathways significantly linked to oral carcinoma progression, including PI3K-AKT (hsa04151), MicroRNAs in cancer (hsa05206), Proteoglycans in cancer (hsa05205) and EGFR resistance (hsa01521) (Supplementary Fig. 15, Supplementary Table 4). Node size indicated gene involvement, while colour represented enrichment significance (− log_10_FDR). These findings suggest that oxidative stress response, apoptosis regulation, and key signalling pathways underlie the anti-oral cancer potential of polyphenols. Network pharmacology confirmed target-protein interactions and highlighted associations with insulin resistance, reactive oxygen species, and apoptosis, supporting their therapeutic relevance^69^. The network analysis supported a systems-level mechanism rather than a single-target effect by revealing multi-target interactions compatible with the observed in vitro bioactivities.

#### Analysis of overlapped target-pathway and target-biological process (BP) networks

Interaction networks based on 20 pathways, 10 biological processes (BPs), and 251 overlapping targets were analysed to elucidate the mechanism of polyphenols against oral carcinoma. Hub targets identified included EGFR (15 pathways, 19 BPs), AKT1 (20 pathways, 19 BPs), TNF–α (17 pathways, 16 BPs), TP53 (16 pathways, 15 BPs), and STAT3 (9 pathways, 14 BPs). Molecular docking showed strong interactions of all the polyphenols especially quercetin pentoside (− 11.2 kcal/mol) and resveratrol dimer (− 11.1 kcal/mol) compared to standard positive control MK-2206. These findings demonstrated that polyphenols can reduce the symptoms of oral carcinoma by targeting a variety of possible targets, including AKT1, EGFR, TP53, TNF-α, and STAT3.The top 5 genes were selected based on its degree and number of pathway associated it (Supplementary Tables 3 and Supplementary Figs. 16–22).These data validate that polyphenols act via multi-target mechanisms, regulating oxidative stress, apoptosis, and carcinoma-related pathways, supporting their therapeutic potential against oral carcinoma and relevance in dental health protection.In previous studies, the identified AKT1 as a core target of quercetin in OSCC, implicating suppression of the PI3K/AKT pathway as a key anticancer mechanism^13^. Earlier computational studies have shown that quercetin act as effective AKT1 binders, including interaction at allosteric or ATP-binding regions, thereby stabilizing the inactive conformation of the kinase^70^. The standard positive control, MK-2206, is a selective allosteric AKT1 inhibitor that efficiently suppresses AKT signaling in oral carcinoma^71^.

The network pharmacology and molecular docking analyses are predictive tools and do not provide direct experimental confirmation of target activity. To address this concern, we have to emphasize that our experimental results (antioxidant, antimicrobial, cytotoxicity, and cytokine suppression assays) provide preliminary biological validation of the polyherbal extract’s activity, supporting the relevance of the predicted targets in the in-silico data. This integrated approach is consistent with well-established network pharmacology principles, according to which mechanistic insight is guided by computational predictions but requires experimental confirmation for validation^72^. The generalizability and transferability of polyherbal methods depend heavily on biological heterogeneity. Network pharmacology is strengthened by combining system-level predictions with experimental validation. Even in noisy data, advances in domain adaptation, including bilateral adaptation networks, enhance transferability and lessen bias^73^. While open-set approaches allow handling of both known and unknown targets, consistent with the multi-target character of polyherbal formulations^74^, universal domain adaptation improves generalization across various datasets through invariant feature learning^75^. The polyherbal extract consistently outperformed untreated controls and displayed modest efficacy in comparison to established references, according to comparative assessments spanning experimental and computational assays. The identified biological effects are more reliable due to these compared trends across independent experiments.

From a translational and real-world perspective, the results of this study offer a scientific basis for creating polyherbal formulations may be supplemental or alternatives for oral carcinoma, especially in situations when affordable and easily accessible treatments are required. Rational formulation design is made possible by the identification of important molecular targets and signaling pathways, which also facilitate the informed advancement of preclinical and clinical evaluation. This method bridges the gap between empirical herbal use and contemporary drug development concepts and improves the practical application of traditional medicine by combining experimental validation with network pharmacology.

## Conclusion

This study demonstrated that the polyherbal leaf mixture of *P. amboinicus*,* O. tenuiflorum*,* M. piperita*,* T. foenum-graecum*, and *A. indica* is rich in bioactive compounds including carbohydrates, amino acids, alkaloids, saponins, glycosides, tannins, bioflavonoids, and polyphenols. The methanolic extract showed superior antioxidant, antimicrobial, anticarcinoma, and anti-inflammatory activities compared to other solvent extracts. GC-MS, UPLC, NMR, and UV analyses confirmed the presence and structural details of key phytonutrients, supporting their traditional medicinal use.Network pharmacology and molecular docking identified most of the compound has strong inhibitors especially quercetin pentoside and resveratrol dimer of AKT1, highlighting their potential as anti-oral cancer. Overall, the findings suggest that polyphenols from this polyherbal mixture may be serving as candidates for oral carcinoma and merit further pharmacological investigation.

## Electronic Supplementary Material

Below is the link to the electronic supplementary material.

Supplementary material 1

Supplementary material 2

## Data Availability

The data are available on request from the authors.
