# Supplementary material for: Development & assessment of polyherbal extracts for treating oral cancer by integrating phytochemistry, bioactivities, and network pharmacology
Source: Sci Rep. 2026 Jul 10;16:21633. doi: 10.1038/s41598-026-53348-z (PMC13354783; doi:10.1038/s41598-026-53348-z)
Supplement: Supplementary file 1 — Supplementary material 1 [file 41598_2026_53348_MOESM1_ESM.doc]

**Supplementary Tables**

**Supplementary table 1:** Phytochemical Screening of the Polyherbal Mixture

| **S. No** | **Phytochemicals** | **Acetone** | **Chloroform** | **Methanol** | **Ethyl Acetate** | **Water** |
| --- | --- | --- | --- | --- | --- | --- |
| 1 | Carbohydrates | ++ | +++ | +++ | +++ | ++ |
| 2 | Fatty Acids | – | – | – | – | – |
| 3 | Proteins | ++ | +++ | +++ | +++ | ++ |
| 4 | Amino Acids | ++ | +++ | ++ | ++ | ++ |
| 5 | Saponins | – | +++ | +++ | – | ++ |
| 6 | Tannins | ++ | +++ | +++ | ++ | ++ |
| 7 | Carotenoids | – | – | – | – | – |
| 8 | Flavonoids | +++ | +++ | +++ | ++ | +++ |
| 9 | Alkaloids | – | – | +++ | ++ | – |
| 10 | Glycosides | +++ | – | ++ | – | ++ |
| 11 | Polyphenols | ++ | +++ | +++ | ++ | ++ |

Note: “–” indicates absence; “++” indicates moderate presence; “+++” indicates strong presence of the respective phytochemical.

**Supplementary table 2:** Zone of Inhibition (in mm) Against Pathogenic Microorganisms by Different Extracts of the Polyherbal Mixture

| **Organism** | **Extract Concentration (µg/mL)** | **Methanol** | **Acetone** | **Ethyl Acetate** | **Standard (500 µg/mL)** |
| --- | --- | --- | --- | --- | --- |
| **S. mutans** | 250 | 10.33 ± 0.57** | 9.20 ± 0.50** | 8.36 ± 0.60** | 17.33 ± 1.50** |
|  | 375 | 11.00 ± 1.00** | 10.00 ± 0.50** | 9.00 ± 1.00** |  |
|  | 500 | 12.66 ± 1.15** | 11.50 ± 1.00** | 10.44 ± 1.00** |  |
| **S. aureus** | 250 | 18.00 ± 1.00** | 15.50 ± 1.00** | 13.20 ± 1.00** | 20.33 ± 0.57** |
|  | 375 | 20.00 ± 1.00** | 16.75 ± 0.50** | 14.20 ± 1.00** |  |
|  | 500 | 21.33 ± 0.57** | 18.00 ± 0.27** | 16.55 ± 0.30** |  |
| **A. viscosus** | 250 | 17.00 ± 1.00** | 14.00 ± 1.00** | 12.00 ± 1.00** | 20.33 ± 0.57** |
|  | 375 | 20.00 ± 1.00** | 15.50 ± 0.50** | 13.50 ± 1.00** |  |
|  | 500 | 20.67 ± 0.57** | 16.87 ± 0.50** | 14.36 ± 0.20** |  |
| **C. albicans** | 250 | 9.67 ± 1.52** | 7.27 ± 0.55** | 6.00 ± 1.00** | 12.33 ± 0.57** |
|  | 375 | 11.67 ± 1.52** | 8.57 ± 1.00** | 7.20 ± 0.58** |  |
|  | 500 | 14.00 ± 1.00** | 10.00 ± 1.00** | 9.50 ± 0.50** |  |

Note: Values are expressed as mean ± standard deviation; p < 0.01 indicates statistical significance compared to control.

**Supplementary table 3:** The 10 biological processes associated with overlapped targets

| **Pathway** | **GO Id** | **Genes** |
| --- | --- | --- |
| Response to peptide hormone | GO:0043434 | SRC/ITGB3/KIT/PDGFRB/MAPK14/HRAS/PTPRA/MDM2/KDR/PARP1/ACE/PPARA/BRAF/PIK3CA/FGFR3/PIK3R1/MTOR/CA2/PTGS2/PRKCA/CYP1B1/EGFR/IGF1R/FLT3/GSK3B/PTK2/MAPK1/CCNA2/SLC2A1/AGTR1/RAF1/RPS6KB1/CTSK/EPHA2/CSK/EPHB2/EPHA4/TYRO3/PTPN1/NFE2L2/STAT3/MET/ERBB2/RELA/AKT1/PPARG/TRPV1/JAK1/ROCK1/OXTR/CSF1R/PRKDC/JAK2/HDAC9/ALK/AXL |
| Cellular response to peptide hormone stimulus | GO:0071375 | SRC/ITGB3/KIT/PDGFRB/HRAS/PTPRA/MDM2/KDR/PARP1/ACE/PIK3CA/FGFR3/PIK3R1/MTOR/CA2/PRKCA/CYP1B1/EGFR/IGF1R/FLT3/GSK3B/PTK2/MAPK1/CCNA2/AGTR1/RAF1/RPS6KB1/EPHA2/CSK/EPHB2/EPHA4/TYRO3/PTPN1/NFE2L2/STAT3/MET/ERBB2/RELA/AKT1/PPARG/JAK1/ROCK1/CSF1R/PRKDC/JAK2/HDAC9/ALK/AXL |
| Positive regulation of phosphorus metabolic process | GO:0010562 | SRC/ITGB3/KIT/ADRB2/PDGFRB/HRAS/MAP2K1/KDR/ERN1/HDAC6/PPARA/BRAF/FGFR3/MTOR/NOX4/TNF/VCP/TP53/APP/EGFR/FLT3/PIM1/SYK/PTK2/PLK1/MMP9/PIK3CG/ADAM17/VEGFA/MAPK1/RAF1/EPHA4/ITGA5/PTPN1/TLR4/CAMKK2/ERBB2/PIN1/CCND1/AKT1/PSEN1/ROCK1/CSF1R/NOD2/AR/P2RX7/HIF1A/JAK2/MIF |
| Positive regulation of phosphate metabolic process | GO:0045937 | SRC/ITGB3/KIT/ADRB2/PDGFRB/HRAS/MAP2K1/KDR/ERN1/HDAC6/PPARA/BRAF/FGFR3/MTOR/NOX4/TNF/VCP/TP53/APP/EGFR/FLT3/PIM1/SYK/PTK2/PLK1/MMP9/PIK3CG/ADAM17/VEGFA/MAPK1/RAF1/EPHA4/ITGA5/PTPN1/TLR4/CAMKK2/ERBB2/PIN1/CCND1/AKT1/PSEN1/ROCK1/CSF1R/NOD2/AR/P2RX7/HIF1A/JAK2/MIF |
| Epithelial cell proliferation | GO:0050673 | ITGB3/KIT/HRAS/AKT3/MAP2K1/PIK3CB/KDR/ERN1/HDAC6/IGFBP3/PIK3CD/ITGA4/XDH/TNF/ALOX5/PRKCA/EGFR/ADAM17/F3/VEGFA/MAPK1/NRAS/AGTR1/RAF1/GLI1/BCL2L1/EPHA2/CXCR3/STAT3/CTNNB1/ERBB2/ESR1/CDK4/CCND1/AKT1/PSEN1/PPARG/CCR3/SMO/NOD2/PRKDC/AR/HIF1A/SCN5A/TACR1/CDK6/MMP12/BAD |
| Vascular endothelial growth factor receptor signaling pathway | GO:0048010 | SRC/ITGB3/KIT/PDGFRB/MAPK14/KDR/FGFR3/EGFR/IGF1R/FLT3/PTK2/PGF/VEGFA/FYN/EPHA2/EPHB2/EPHA4/TYRO3/ITGA5/PTPN1/MET/ERBB2/CSF1R/HIF1A/ALK/AXL |
| Cellular response to vascular endothelial growth factor stimulus | GO:0035924 | KIT/PDGFRB/MAPK14/ITGB1/PIK3CB/KDR/ERN1/PIK3CA/PIK3CD/FGFR3/XDH/EGFR/IGF1R/FLT3/PGF/VEGFA/EPHA2/EPHB2/EPHA4/TYRO3/MET/ERBB2/RELA/AKT1/CSF1R/ALK/AXL |
| Response to amyloid-beta | GO:1904645 | ADRB2/ICAM1/PARP1/HDAC2/ITGA4/TNF/APP/ABCC1/IGF1R/SYK/GSK3B/MMP13/MMP3/MMP9/MMP2/FYN/EPHB2/EPHA4/TLR4/PSEN1/MMP12 |
| Positive regulation of phosphorylation | GO:0042327 | SRC/ITGB3/KIT/ADRB2/PDGFRB/HRAS/MAP2K1/KDR/ERN1/HDAC6/BRAF/FGFR3/MTOR/NOX4/TNF/TP53/APP/EGFR/FLT3/PIM1/SYK/PTK2/PLK1/MMP9/PIK3CG/ADAM17/VEGFA/MAPK1/RAF1/EPHA4/ITGA5/PTPN1/TLR4/CAMKK2/ERBB2/PIN1/CCND1/AKT1/CSF1R/NOD2/AR/JAK2/MIF |
| Response to xenobiotic stimulus | GO:0009410 | TOP1/ALDH2/SRC/ITGB3/CDK1/MDM2/ACE/CASP3/BRAF/HDAC2/PTGS2/TNF/TP53/CYP1B1/CA9/EGFR/ABCB1/ABCC1/MMP2/MMP7/ADAM17/HSP90AA1/TYMS/CHEK2/RPS6KB1/NFE2L2/AHR/CTNNB1/CYP1A1/ADA/RELA/CDK4/CCND1/TSPO/OXTR/P2RX7/SLC6A4/PCNA/NOS2/NQO1/AKR1C1/BCL2/CBR1 |

**Supplementary table 4:** The 20 pathways associated with overlapped targets.

| **#Term ID** | **Term description** | **Matching proteins in the network (labels)** |
| --- | --- | --- |
| hsa05215 | Prostate cancer pathway | AKT1, AKT3, AR, BAD, BCL2, BRAF, CCND1, CTNNB1, EGFR, ERBB2, GSK3B, HRAS, HSP90AA1, IGF1R, MAP2K1, MAPK1, MDM2, MMP3, MMP9, MTOR, NRAS, PDGFRB, PIK3CA, PIK3CB, PIK3CD, PIK3R1, RAF1, RELA, TP53 |
| hsa01521 | EGFR tyrosine kinase inhibitor resistance pathway | AKT1, AKT3, AXL, BAD, BCL2, BCL2L1, BRAF, EGFR, ERBB2, FGFR3, GSK3B, HRAS, IGF1R, JAK1, JAK2, KDR, MAP2K1, MAPK1, MET, MTOR, NRAS, PDGFRB, PIK3CA, PIK3CB, PIK3CD, PIK3R1, PRKCA, RAF1, RPS6KB1, SRC, STAT3, VEGFA |
| hsa05205 | Proteoglycans in cancer pathway | AKT1, AKT3, BRAF, CASP3, CCND1, CTNNB1, EGFR, ERBB2, ESR1, HIF1A, HRAS, IGF1R, ITGA5, ITGB1, ITGB3, KDR, MAP2K1, MAPK1, MAPK14, MDM2, MET, MMP2, MMP9, MTOR, NRAS, PIK3CA, PIK3CB, PIK3CD, PIK3R1, PRKCA, PTK2, RAF1, ROCK1, RPS6KB1, SMO, SRC, STAT3, TLR4, TNF, TP53, VEGFA |
| hsa04151 | PI3K-Akt signaling pathway | AKT1, AKT3, BAD, BCL2, BCL2L1, CCND1, CDK4, CDK6, CSF1R, EGFR, EPHA2, ERBB2, FGFR3, FLT3, GSK3B, HRAS, HSP90AA1, IGF1R, ITGA4, ITGA5, ITGB1, ITGB3, JAK1, JAK2, KDR, KIT, MAP2K1, MAPK1, MDM2, MET, MTOR, NRAS, PDGFRB, PGF, PIK3CA, PIK3CB, PIK3CD, PIK3CG, PIK3R1, PRKCA, PTK2, RAF1, RELA |
| hsa01522 | Endocrine resistance pathway | AKT1, AKT3, BAD, BCL2, BRAF, CCND1, CDK4, EGFR, ERBB2, ESR1, HRAS, IGF1R, MAP2K1, MAPK1, MAPK14, MDM2, MMP2, MMP9, MTOR, NRAS, PIK3CA, PIK3CB, PIK3CD, PIK3R1, PTK2, RAF1, RPS6KB1, SRC, TP53 |
| has04933 | AGE-RAGE signaling pathway in diabetic complications | AGTR1, AKT1, AKT3, BCL2, CASP3, CCND1, CDK4, F3, HRAS, ICAM1, JAK2, MAPK1, MAPK14, MMP2, NOX4, NRAS, PIK3CA, PIK3CB, PIK3CD, PIK3R1, PIM1, PRKCA, RELA, STAT3, TNF, VEGFA |
| hsa05221 | Acute myeloid leukemia pathway | AKT1, AKT3, BAD, BRAF, CCNA2, CCND1, CSF1R, FLT3, HRAS, KIT, MAP2K1, MAPK1, MTOR, NRAS, PIK3CA, PIK3CB, PIK3CD, PIK3R1, PIM1, RAF1, RELA, RPS6KB1, STAT3 |
| hsa04510 | Focal adhesion pathway | AKT1, AKT3, BAD, BCL2, BRAF, CCND1, CTNNB1, EGFR, ERBB2, FYN, GSK3B, HRAS, IGF1R, ITGA4, ITGA5, ITGB1, ITGB3, KDR, MAP2K1, MAPK1, MET, PDGFRB, PGF, PIK3CA, PIK3CB, PIK3CD, PIK3R1, PRKCA, PTK2, RAF1, ROCK1, SRC, VEGFA |
| hsa05161 | Hepatitis B pathway | AKT1, AKT3, BAD, BCL2, BRAF, CASP3, CCNA2, HRAS, JAK1, JAK2, MAP2K1, MAPK1, MAPK14, MMP9, NRAS, PCNA, PIK3CA, PIK3CB, PIK3CD, PIK3R1, PRKCA, RAF1, RELA, SRC, STAT3, TLR4, TNF, TP53 |
| hsa05417 | Lipid and atherosclerosis pathway | AKT1, AKT3, BAD, BCL2, BCL2L1, CASP3, CYP1A1, ERN1, GSK3B, HRAS, HSP90AA1, ICAM1, JAK2, MAPK1, MAPK14, MMP3, MMP9, NFE2L2, NRAS, PIK3CA, PIK3CB, PIK3CD, PIK3R1, PPARG, PRKCA, PTK2, RELA, SRC, STAT3, TLR4, TNF, TP53 |
| hsa05219 | Bladder cancer pathway | AKT1, BRAF, CCND1, CDK4, EGFR, ERBB2, FGFR3, HRAS, MAP2K1, MAPK1, MDM2, MMP2, MMP9, NRAS, RAF1, SRC, TP53, VEGFA |
| hsa05212 | Pancreatic cancer pathway | AKT1, AKT3, BAD, BCL2L1, BRAF, CCND1, CDK4, CDK6, EGFR, ERBB2, JAK1, MAP2K1, MAPK1, MTOR, PIK3CA, PIK3CB, PIK3CD, PIK3R1, RAF1, RELA, RPS6KB1, STAT3, TP53, VEGFA |
| hsa05167 | Kaposi sarcoma-associated herpesvirus infection pathway | AKT1, AKT3, CASP3, CCND1, CCR3, CDK4, CDK6, CTNNB1, GSK3B, HIF1A, HRAS, ICAM1, JAK1, JAK2, MAP2K1, MAPK1, MAPK14, MTOR, NRAS, PIK3CA, PIK3CB, PIK3CD, PIK3CG, PIK3R1, PTGS2, RAF1, RELA, SRC, STAT3, SYK, TP53, VEGFA |
| hsa05165 | Human papillomavirus infection pathway | AKT1, AKT3, BAD, CASP3, CCNA2, CCND1, CDK4, CDK6, CTNNB1, EGFR, GSK3B, HDAC2, HRAS, ITGA4, ITGA5, ITGB1, ITGB3, JAK1, MAP2K1, MAPK1, MDM2, MTOR, NRAS, PDGFRB, PIK3CA, PIK3CB, PIK3CD, PIK3R1, PSEN1, PTGS2, PTK2, RAF1, RELA, RPS6KB1, TNF, TP53, VEGFA |
| hsa05230 | Central carbon metabolism in cancer pathway | AKT1, AKT3, EGFR, ERBB2, FGFR3, FLT3, HIF1A, HRAS, KIT, MAP2K1, MAPK1, MET, MTOR, NRAS, PDGFRB, PIK3CA, PIK3CB, PIK3CD, PIK3R1, RAF1, SLC2A1, TP53 |
| hsa05163 | Human cytomegalovirus infection pathway | AKT1, AKT3, EGFR, ERBB2, FGFR3, FLT3, HIF1A, HRAS, KIT, MAP2K1, MAPK1, MET, MTOR, NRAS, PDGFRB, PIK3CA, PIK3CB, PIK3CD, PIK3R1, RAF1, SLC2A1, TP53 |
| hsa05206 | MicroRNAs in cancer pathway | AKT1, ABCB1, ABCC1, BCL2, CASP3, CDK6, CYP1B1, EGFR, ERBB2, FGFR3, HDAC2, HRAS, ITGA5, ITGB3, MAP2K1, MAPK1, MDM2, MET, MMP9, MTOR, NRAS, PDGFRB, PIK3CA, PIK3CB, PIK3CD, PIK3R1, PIM1, PRKCA, PTGS2, RAF1, ROCK1, STAT3, TP53, VEGFA |
| hsa05203 | Viral carcinogenesis pathway | AKT1, BAD, CASP3, CCNA2, CCND1, CCR3, CDK1, CDK4, CDK6, HDAC2, HDAC6, HDAC9, HRAS, JAK1, MAPK1, MDM2, NRAS, PIK3CA, PIK3CB, PIK3CD, PIK3R1, RELA, SRC, STAT3, SYK, TP53 |
| hsa04919 | Thyroid hormone signaling pathway | AKT1, AKT3, BAD, CCND1, CTNNB1, ESR1, GSK3B, HDAC2, HIF1A, HRAS, ITGB3, MAP2K1, MAPK1, MDM2, MTOR, NRAS, PIK3CA, PIK3CB, PIK3CD, PIK3R1, PRKCA, RAF1, SLC2A1, SRC, TP53 |
| hsa04218 | Cellular senescence pathway | AKT1, AKT3, CCNA2, CCND1, CDK1, CDK4, CDK6, CHEK2, HRAS, IGFBP3, MAP2K1, MAPK1, MAPK14, MDM2, MTOR, NRAS, PIK3CA, PIK3CB, PIK3CD, PIK3R1, RAF1, RELA, TP53 |

**Supplementary table 5: Molecular Docking of Polyphenols and top 5 genes**

| **COMPOUNDS** | Force field | **AKT1** | **EGFR** | **TNF** | **TP53** | **STAT3** |
| --- | --- | --- | --- | --- | --- | --- |
| Catechol dimer (Ox1) | 55.66 | -5.2 | -4.9 | -4.8 | -4.8 | -4.3 |
| Feruloyl tyramine 4-O-hexoside | 477.87 | -10.3 | -6.9 | -7.1 | -8.6 | -5.7 |
| Isorhamnetin-3-O-glucoside | 507.59 | -10.4 | -8.0 | -8.6 | -7.6 | -6.3 |
| Myricetin | 256.53 | -9.6 | -7.6 | -7.8 | -7.6 | -6.7 |
| Oleuropein glucoside | 698.99 | -9.5 | -7.4 | -7.1 | -7.7 | -5.5 |
| Puerarin | 644.77 | -10.1 | -8.1 | -7.9 | -7.8 | -6.6 |
| Quercetin [H2O]+ | 234.71 | -9.5 | -7.6 | -7.6 | -7.6 | -6.6 |
| Quercetin pentoside | 872.69 | -11.2 | -8.3 | -8.6 | -8.8 | -7.1 |
| Quercetin rhamnoside-O hexoside | 962.27 | -10.9 | -8.1 | -8.1 | -8.2 | -7.5 |
| Resveratrol dimer | 648.14 | -11.1 | -8.0 | -7.8 | -8.3 | -7.8 |
| Sinapic acid | 213.58 | -6.9 | -5.7 | -5.6 | -5.8 | -4.9 |
| Syringaresinol | 625.30 | -10.1 | -7.0 | -7.3 | -6.8 | -6.6 |
| Vitamin E – Tocopherol | 636.66 | -9.0 | -5.6 | -5.7 | -6.9 | -5.0 |
