# Supplementary material for: Development & assessment of polyherbal extracts for treating oral cancer by integrating phytochemistry, bioactivities, and network pharmacology
Source: Sci Rep. 2026 Jul 10;16:21633. doi: 10.1038/s41598-026-53348-z (PMC13354783; doi:10.1038/s41598-026-53348-z)
Supplement: Supplementary file 2 — Supplementary material 2 [file 41598_2026_53348_MOESM2_ESM.doc]

**Supplementary Figures**


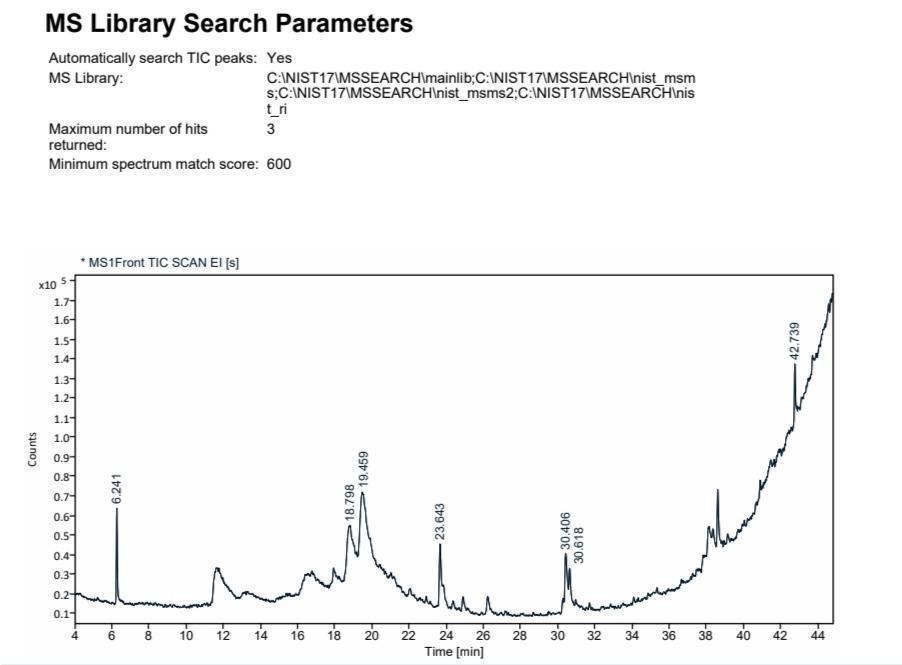


**Supplementaryfigure 1:** GC-MS Spectrum of Methanol extract of the Polyherbal Mixture


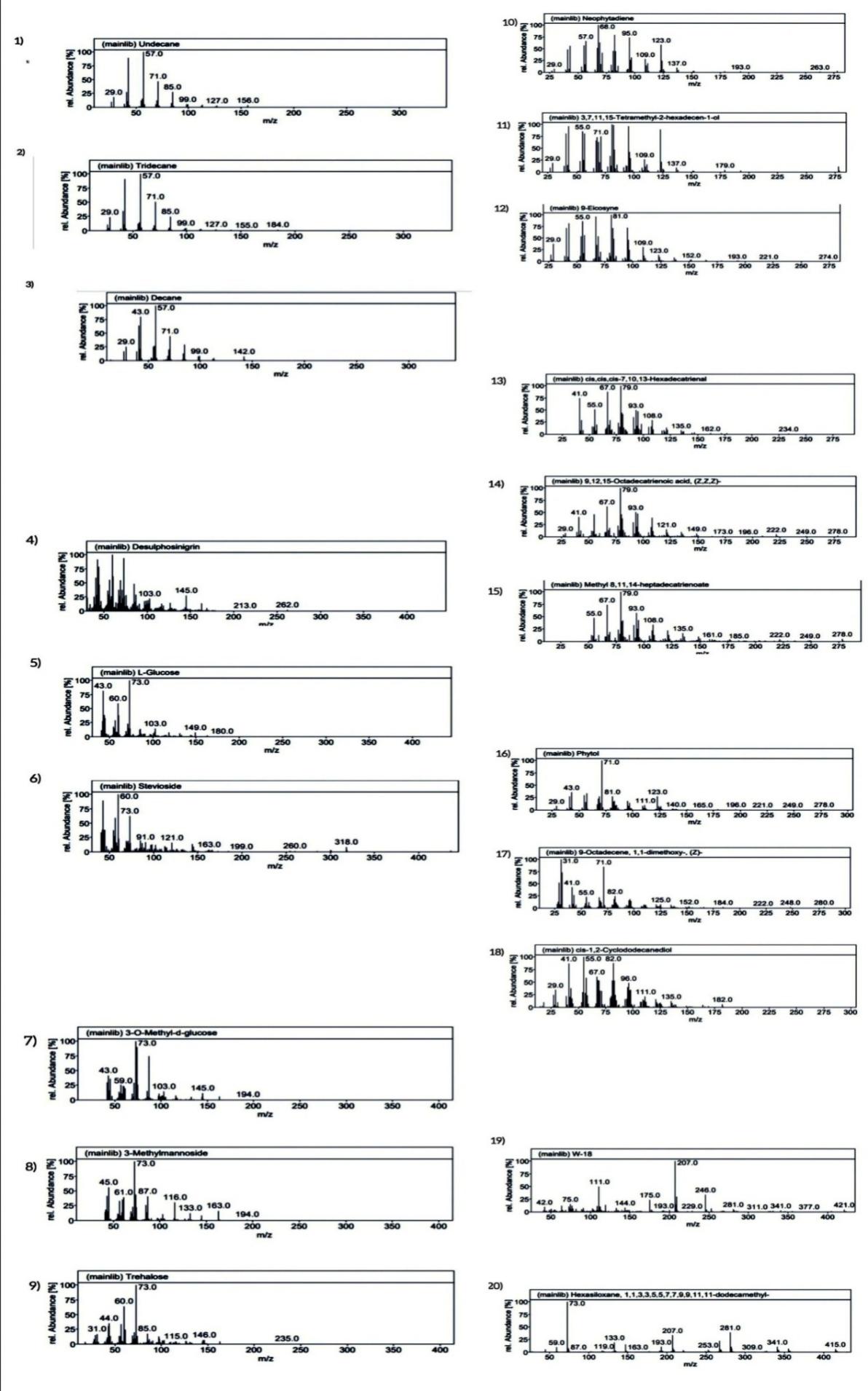


**Supplementary figure 2:** GCMS chromatograms displaying the identified phytonutrients in the polyherbal extract


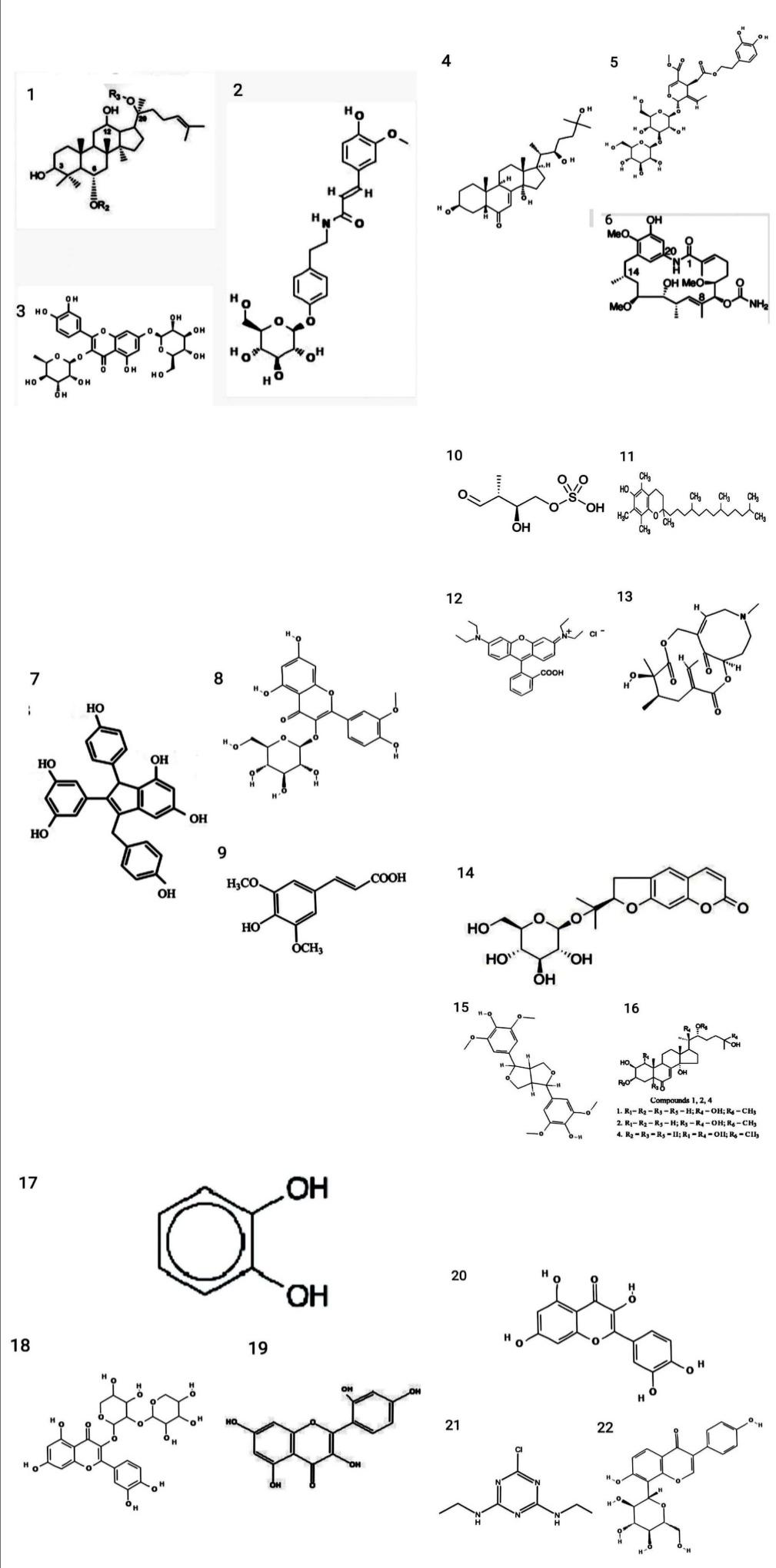


**Supplementary figure 3:** Chemical structure of polyherbal UPLC phytonutrient compounds

**
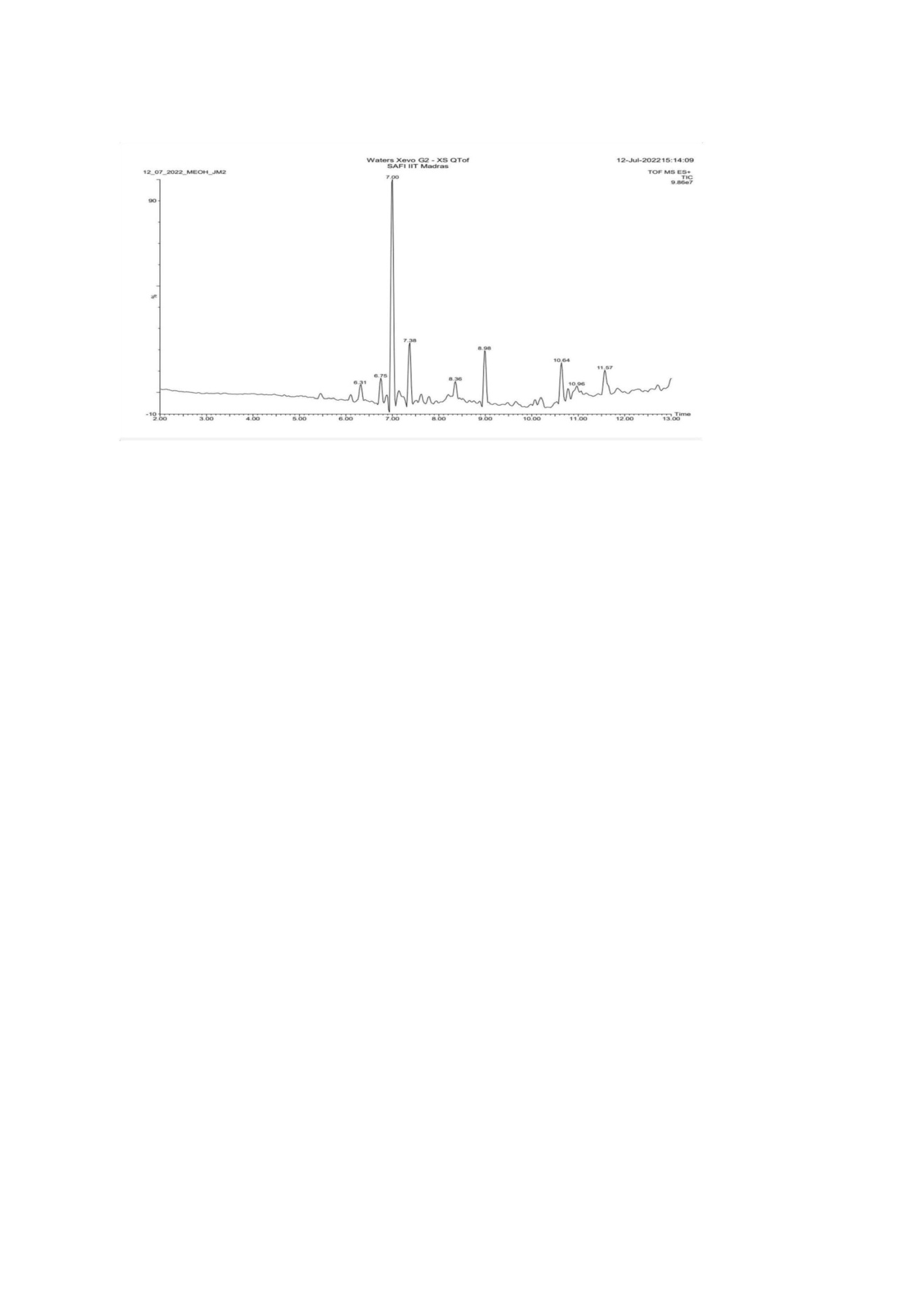
**

**Supplementary figure 4:** UPLC Chromatogram of Methanol extract of the Polyherbal Mixture


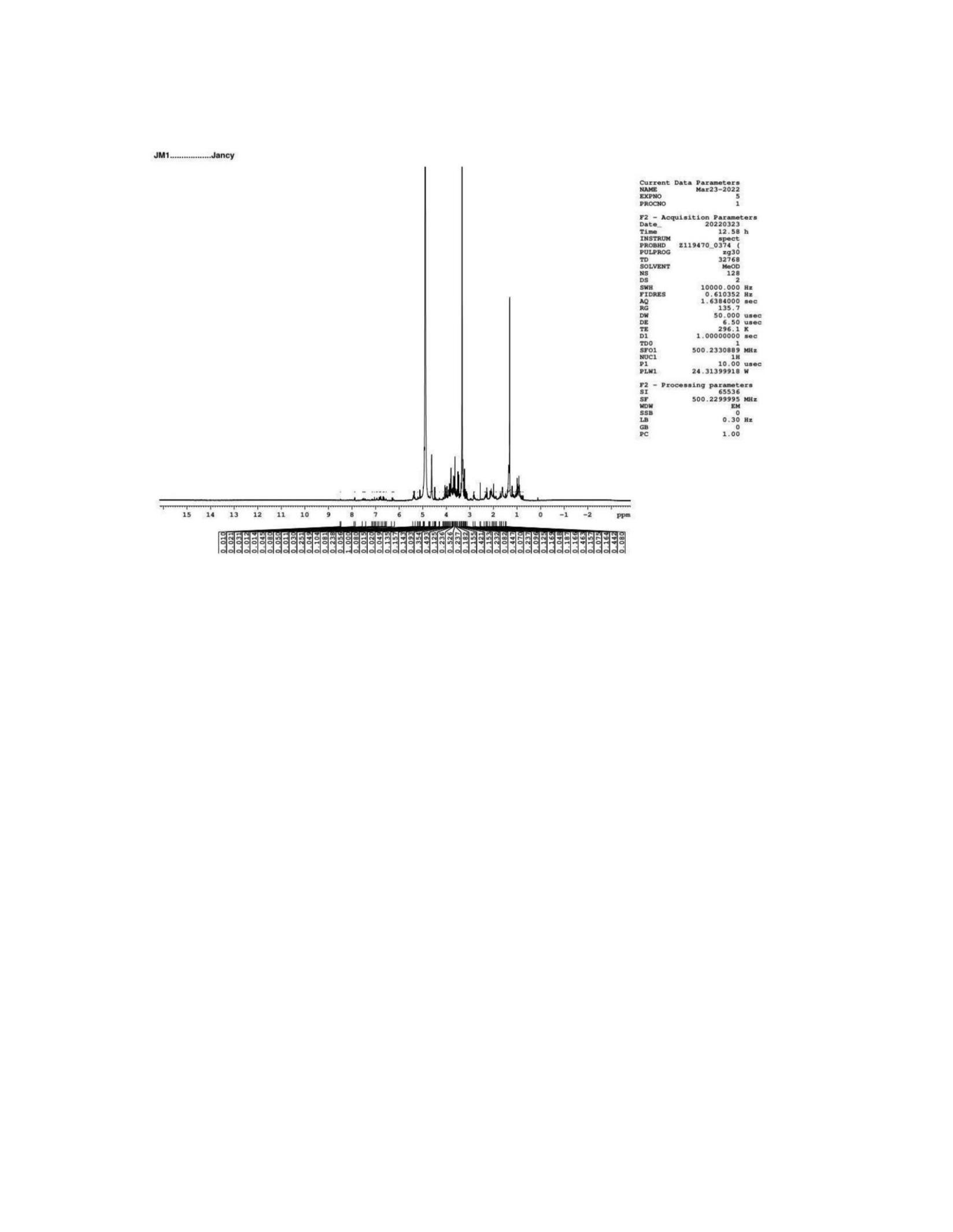


**Supplementary figure5:** NMR Spectrum of Methanol extract of Polyherbal Mixture


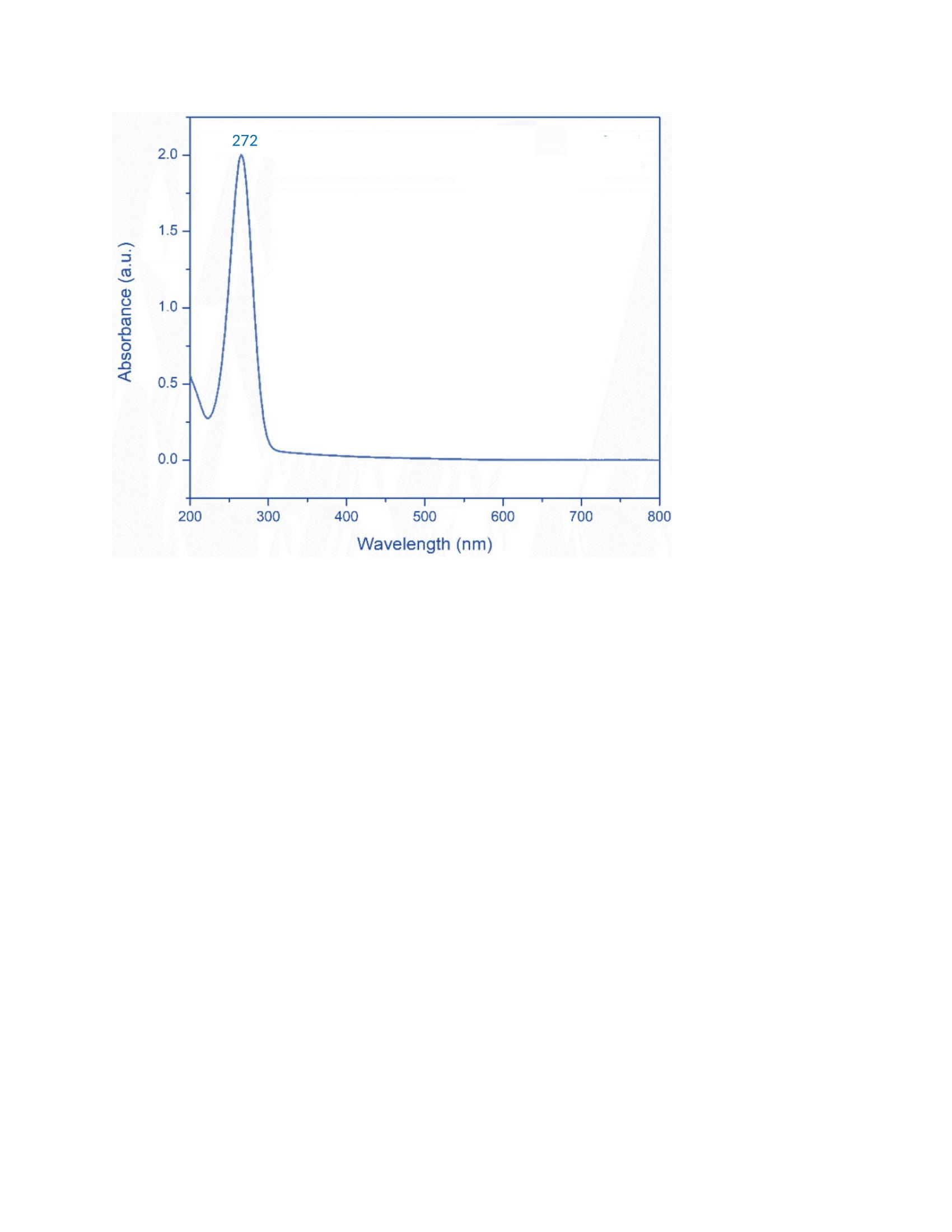


**Supplementary figure 6:** UV-Visible spectroscopy


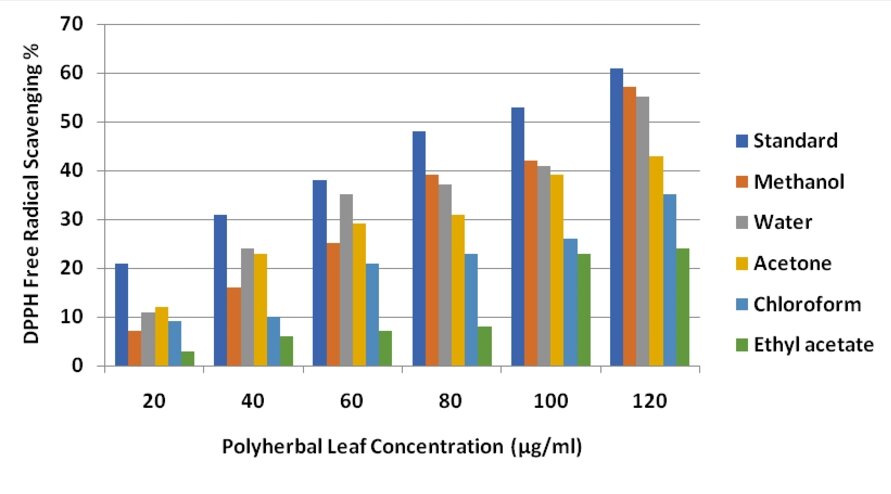


**Supplementary figure 7:** Antioxidant Effect of the different extracts of polyherbal mixture


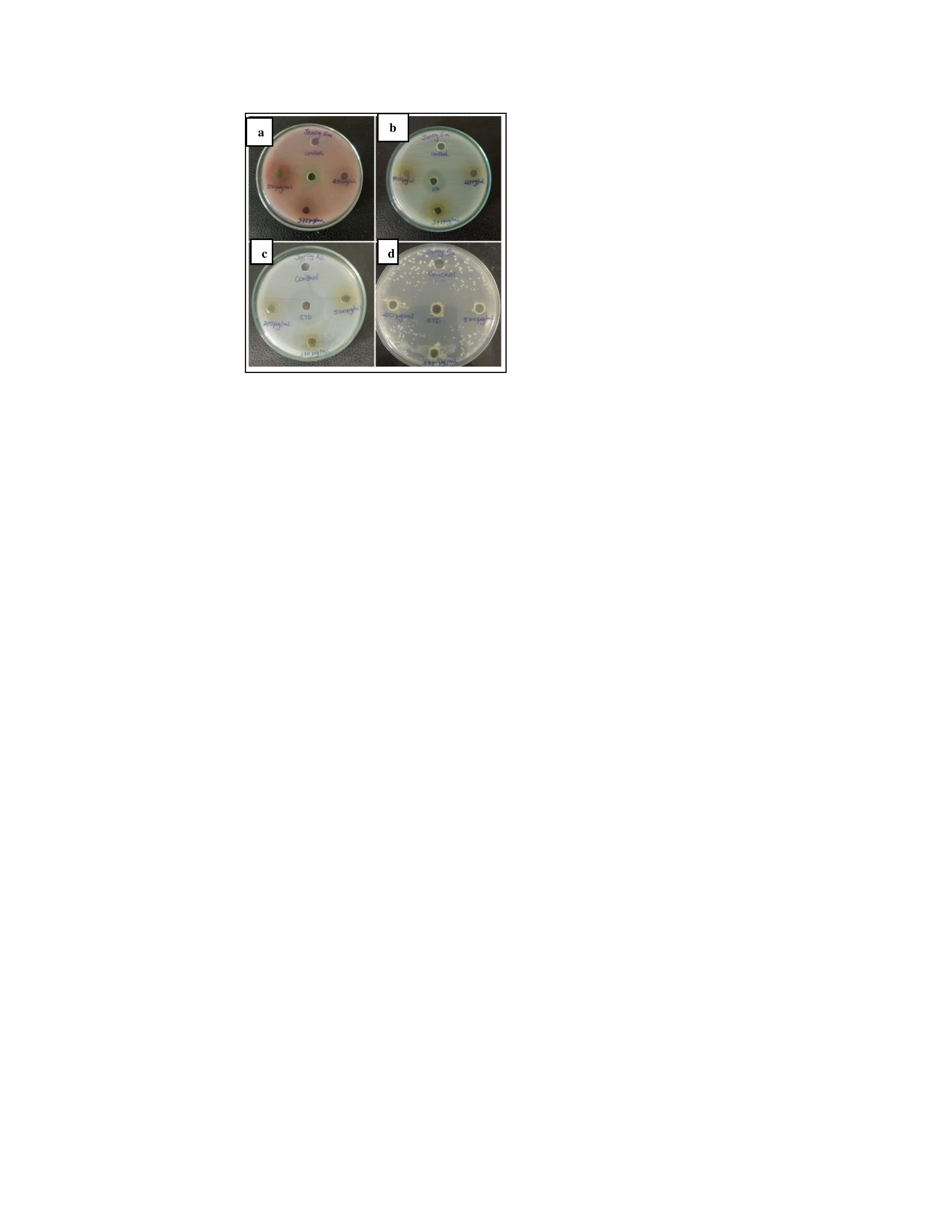


**Supplementary figure 8:** Antimicrobial activity of methanolic polyherbal extract against (a) Streptococcus mutans, (b) Staphylococcus aureus, (c) Actinomyces viscosus (Standard: Tetracycline), and (d) Candida albicans (Standard: Fluconazole).


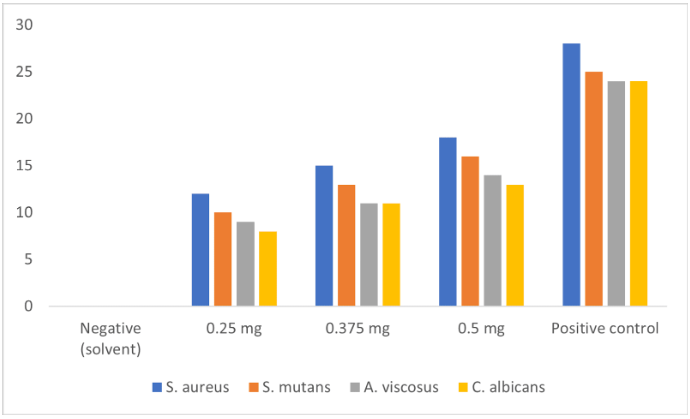


**Supplementary figure 9:** Minimum Inhibitory Concentration (MIC) of methanolic polyherbal extract against (a) Streptococcus mutans, (b) Staphylococcus aureus, (c) Actinomyces viscosus (Standard: Tetracycline), and (d) Candida albicans (Standard: Fluconazole). X- axis- Microbial species and Y-axis - **µg** /well


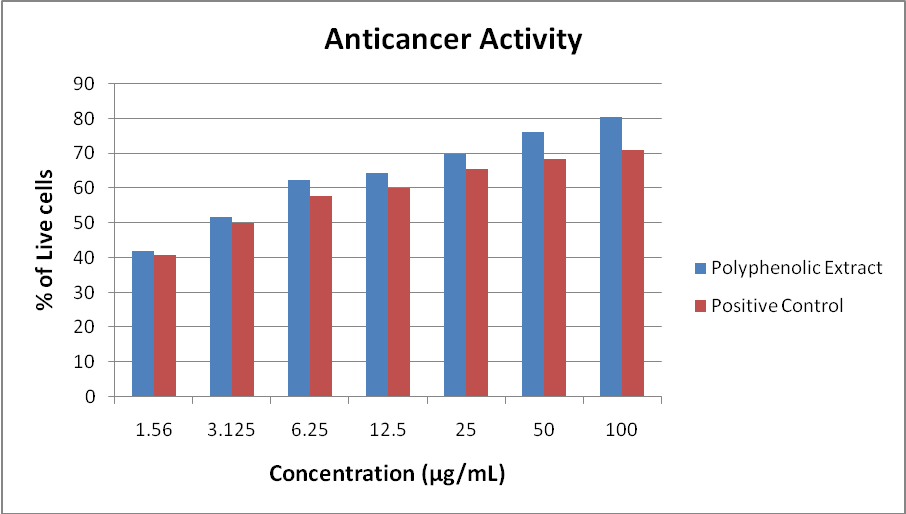


**Supplementary figure 10:** Anticancer Activity of Polyphenolic Extract against Positive Control


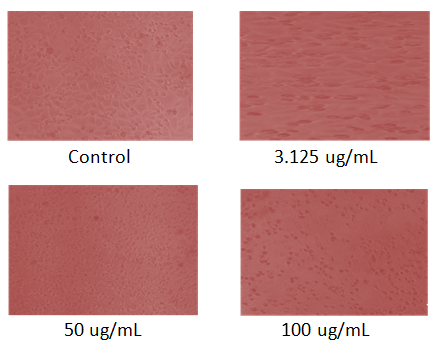


**Supplementary figure 11:** Anticancer Activity of Polyphenolic Extract against Normal Vero Cell line


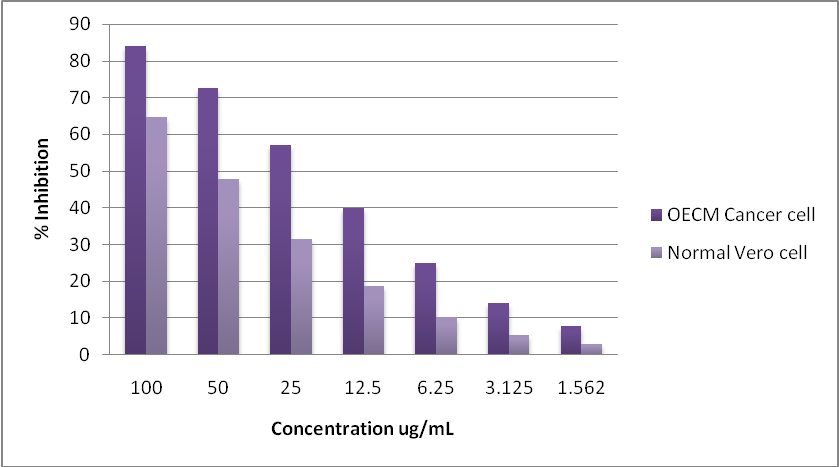


**Supplementary figure 12:** Inhibition of cytotoxic effect of OECM and Normal cell


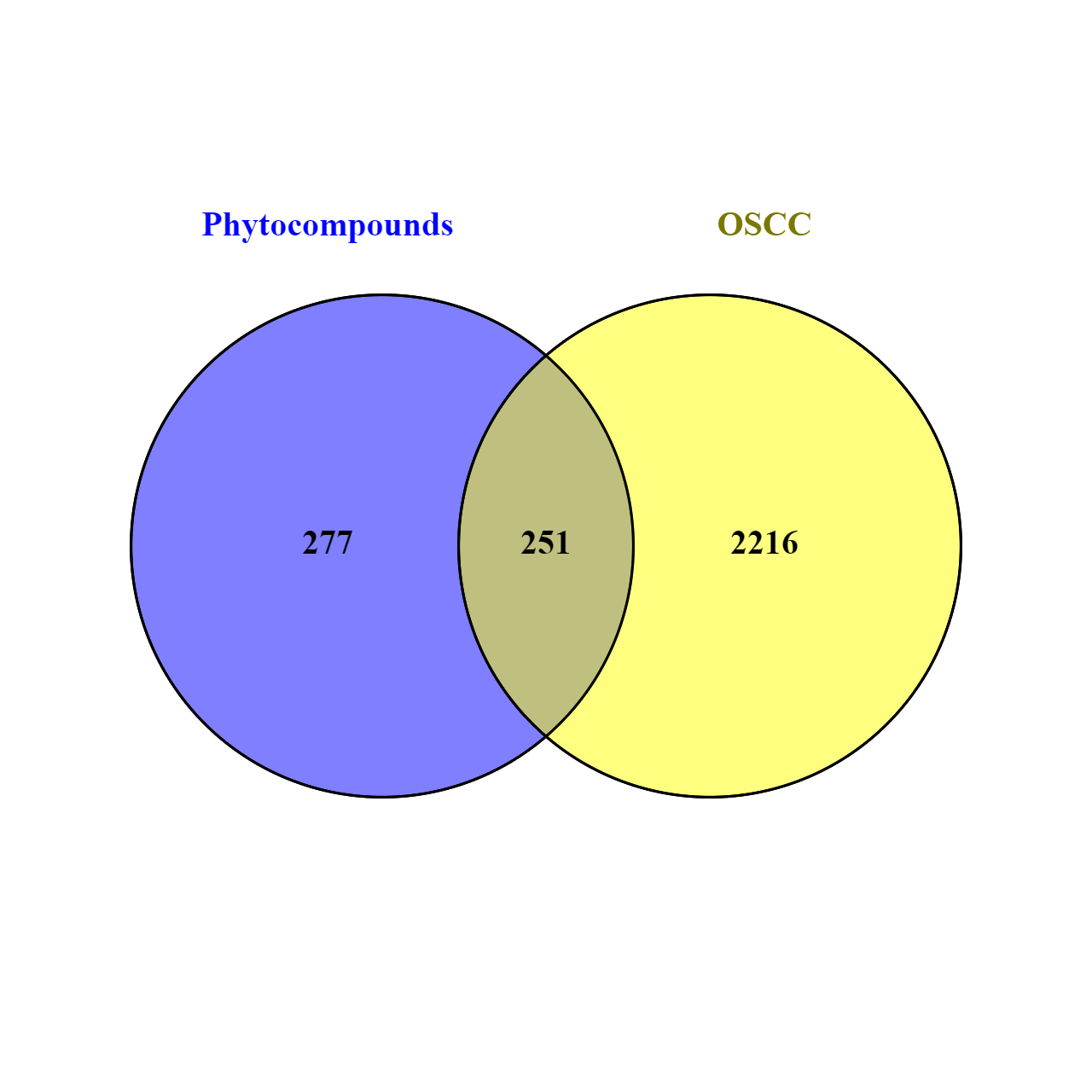


**Supplementary figure 13:** Overlapping Targets of Polyphenols and Oral Cancer


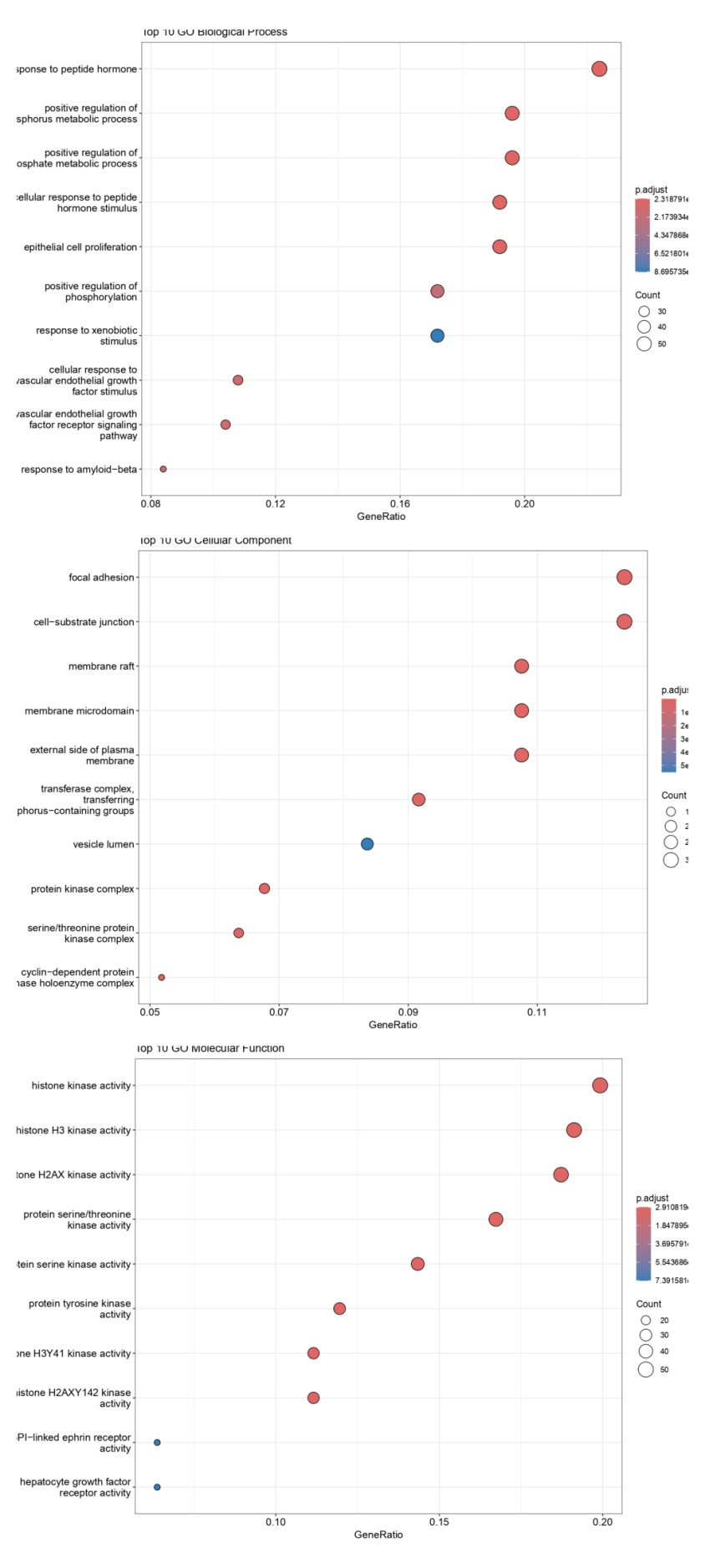


**Supplementary figure 14:** GO Enrichment Analysis of the overlapped targets (Top- Biological Process, Middle- Cellular component and Bottom- Molecular function)


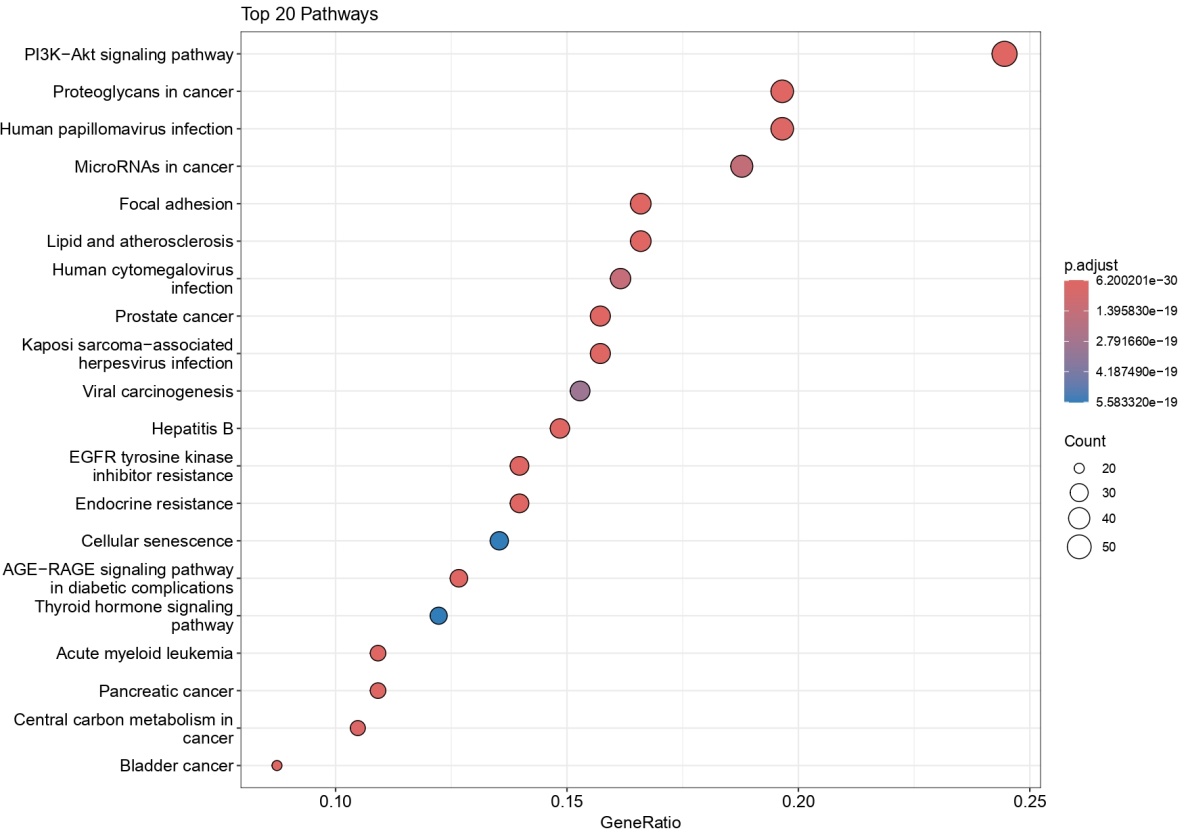


**Supplementary figure 15** The KEGG pathways associated with overlapped targets.

**
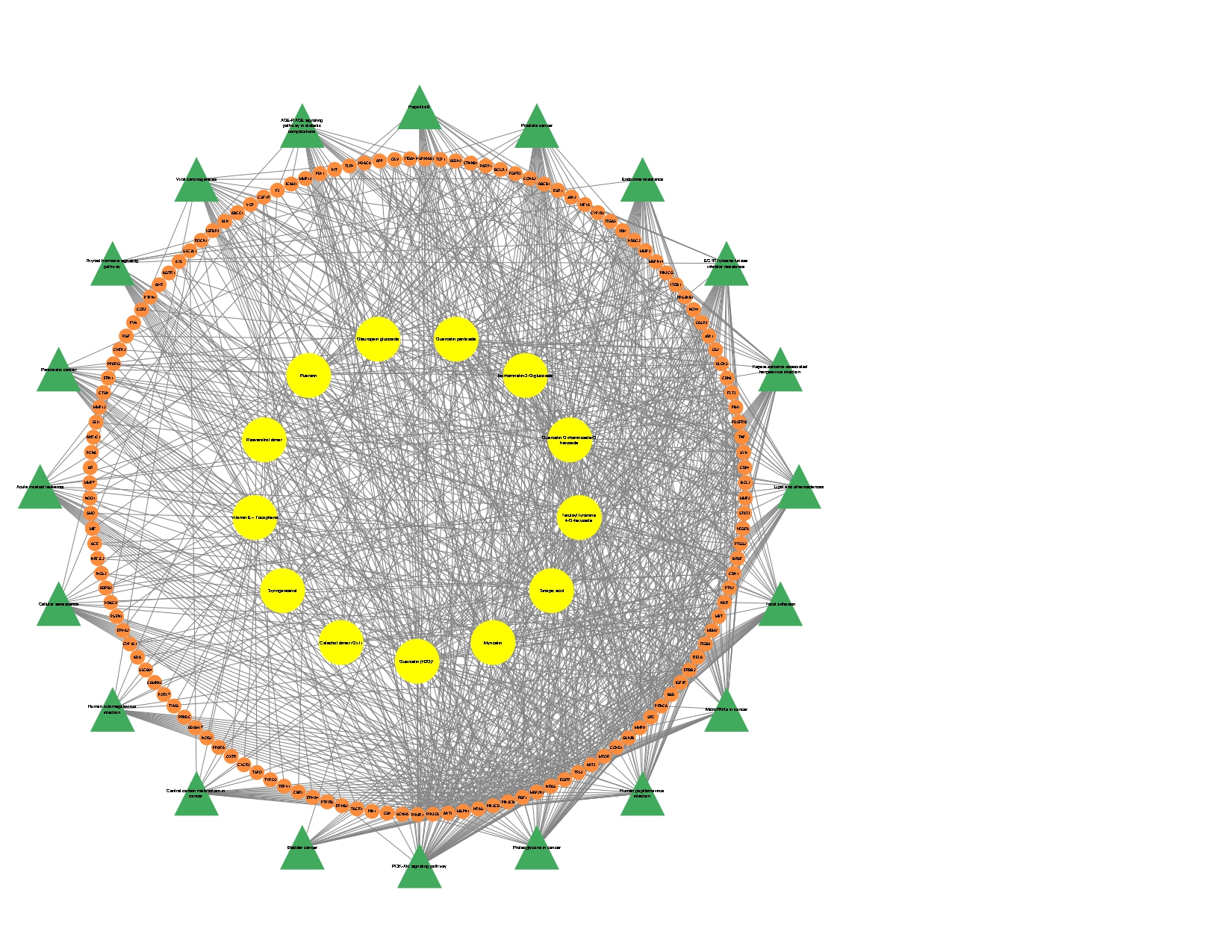
**

**Supplementary figure 16:** Targets and their likely protein molecules interact in a network to cause oral cancer. (Yellow color indicates Polyphenolic compounds, Orange color indicates gene ontology ID’s, Green color indicates specific pathways)


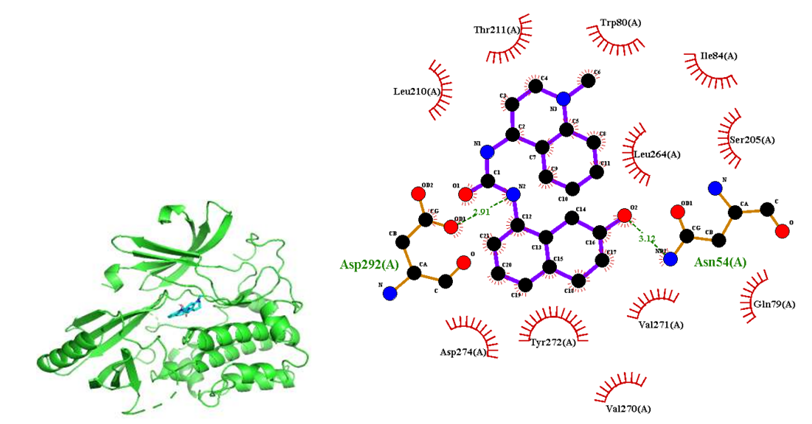


**Supplementary figure 17:** Docked conformations of AKT1 with Positive Control MK-2206

**
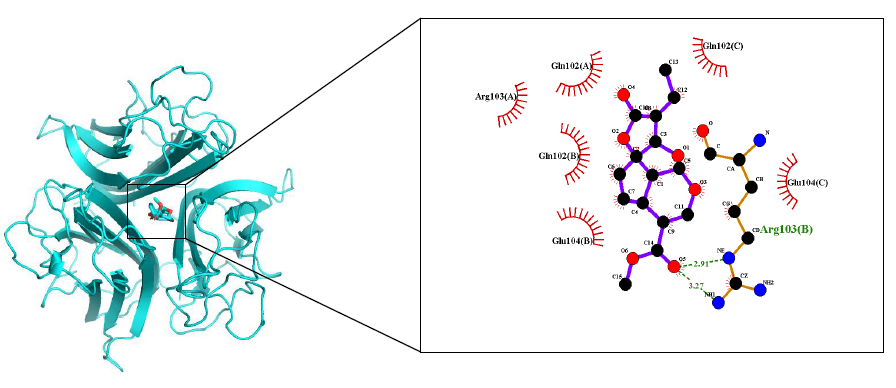
**

**Supplementary figure 18:** Docked conformations of AKT1 with Feruloyl tyramine 4-O-hexoside

**
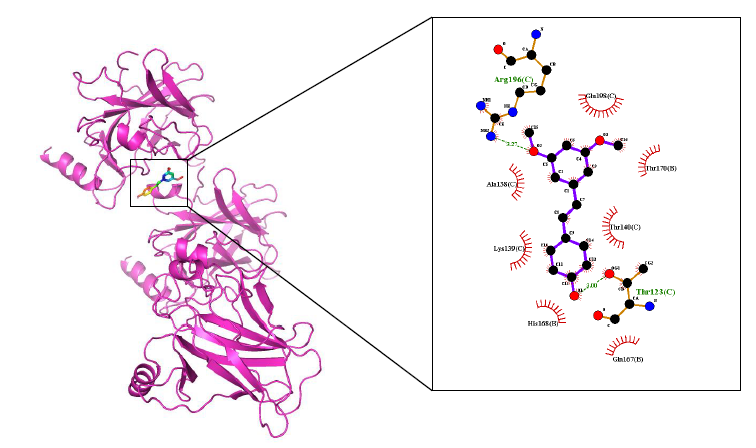
**

**Supplementary figure 19:** Docked conformations of AKT1 with Isorhamnetin-3-O-glucoside


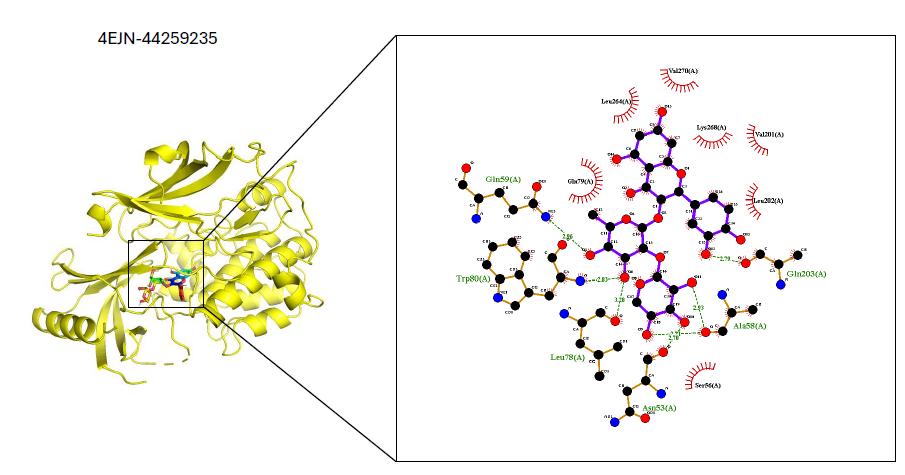


**Supplementary figure 20:** Docked conformations of AKT1 with Quercetin pentoside

**
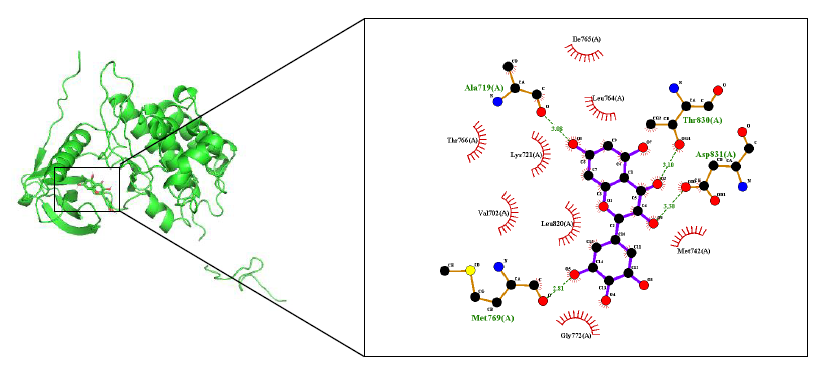
**

**Supplementary figure 21:** Docked conformations of AKT1 with Quercetin rhamnoside-O hexoside

**
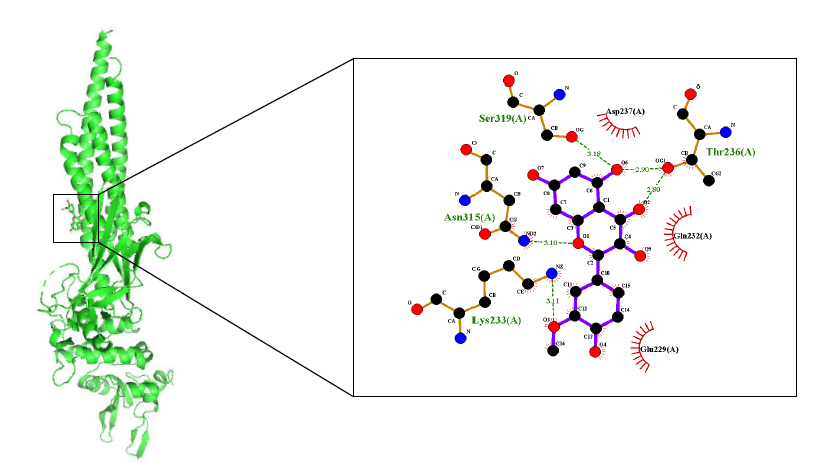
**

**Supplementary figure 22:** Docked conformations of AKT1 with Resveratrol dimer


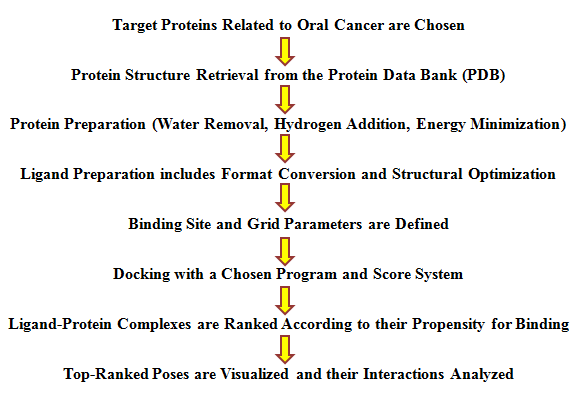


**Supplementary figure 23: Preparation Step of *insilico* studies**
